# Thermally Activated Delayed Fluorescence (TADF) Materials Based on Earth‐Abundant Transition Metal Complexes: Synthesis, Design and Applications

**DOI:** 10.1002/advs.202404866

**Published:** 2024-07-10

**Authors:** Valentina Ferraro, Claudia Bizzarri, Stefan Bräse

**Affiliations:** ^1^ Institute of Organic Chemistry (IOC) Karlsruhe Institute of Technology (KIT) Kaiserstrasse 12 76131 Karlsruhe Germany; ^2^ Institute of Biological and Chemical Systems‐Functional Molecular Systems (IBCS‐FMS) Karlsruhe Institute of Technology (KIT) Kaiserstrasse 12 76131 Karlsruhe Germany

**Keywords:** earth‐abundant, photoconversion materials, sustainable light‐emitting devices, TADF, transition metal complexes

## Abstract

Materials exhibiting thermally activated delayed fluorescence (TADF) based on transition metal complexes are currently gathering significant attention due to their technological potential. Their application extends beyond optoelectronics, in particular organic light‐emitting diodes (OLEDs) and light‐emitting electrochemical cells (LECs), and include also photocatalysis, sensing, and X‐ray scintillators. From the perspective of sustainability, earth‐abundant metal centers are preferred to rarer second‐ and third‐transition series elements, thus determining a reduction in costs and toxicity but without compromising the overall performances. This review offers an overview of earth‐abundant transition metal complexes exhibiting TADF and their application as photoconversion materials. Particular attention is devoted to the types of ligands employed, helping in the design of novel systems with enhanced TADF properties.

## Graphical abstract

Thermally activated delayed fluorescence (TADF) is gaining particular attention for its promising technological application. This review focuses on earth‐abundant transition metal complexes exhibiting TADF and their employment as photoconversion materials, in organic light‐emitting diodes (OLEDs), light‐emitting electrochemical cells (LECs), photocatalysis, sensing, and X‐ray scintillators.

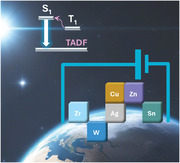

## 1 Introduction

Organometallic emitters based on Ir(III) and Pt(II) complexes are commercially exploited for the preparation of organic light‐emitting diodes (OLEDs) exhibiting internal quantum efficiencies (IQEs) of up to 100%. Owing to the large spin‐orbit coupling (SOC) effect that determines phosphorescence from the first triplet *T*_1_ to the ground state *S*_0_, these elements have attracted great interest. However, the cost of the device is often affected by the low abundance of the starting metal salts on the earth's crust. Despite their impressive performances as red and green emitters, potential environmental contamination during their disposing is another aspect that cannot be ignored.^[^ ^1^ ^]^ Within these sustainability issues, the interest in photoluminescent earth‐abundant transition metal complexes (TMCs) has emerged.^[^ ^2^ ^]^

Performances similar to phosphorescent Ir(III) and Pt(II) complexes were observed in OLEDs based on emitters characterized by thermally activated delayed fluorescence (TADF).^[^ ^1^ ^]^ These promising photoluminescent properties rely on the peculiar exciton harvesting mechanism, as will be further detailed in the following pages. In contrast to purely organic TADF materials, TMCs are extremely versatile in terms of *d*‐block metal centers and ligands, which are often easily accessible. The plethora of possible emission mechanisms and the adaptable design of TMCs enable emission wavelengths to potentially cover the entire visible range of the spectrum. In addition, the nature of the metal center and surrounding ligands allows for both neutral and charged TMCs, making them attractive emitters for OLEDs and light‐emitting electrochemical cells (LECs), respectively.^[^ ^3^ ^]^

In the following pages, earth‐abundant TMCs exhibiting TADF properties will be discussed together with the design principles to achieve these photophysical properties, and their application as photoconversion materials. In particular, examples of TADF materials containing earth‐abundant TMCs will be presented according to the group in the periodic table. Due to the multitude of examples containing Cu(I) as metal center, the complexes will be further divided depending on the type of ligands.

## 2 TADF Principles

As previously stated, materials exhibiting TADF properties are of current interest to be exploited for optoelectronic applications, particularly OLEDs and LECs. TADF was first observed in eosin at the beginning of the Sixties and was therefore termed *E*‐type delayed fluorescence, referring to its first observation.^[^ ^4^ ^]^ The first complex exhibiting TADF properties was described twenty years later when Blasse and McMillin investigated the emission of [Cu(phen)(PPh_3_)_2_]^+^ at variable temperatures.^[^ ^5^ ^]^

**Figure 1:**
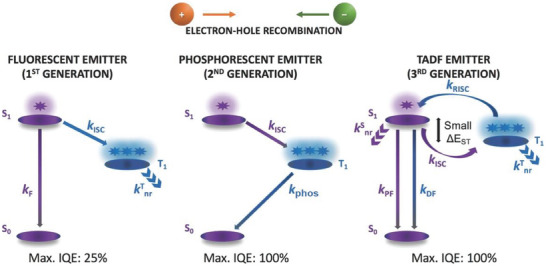
Comparison of emission mechanism in first‐generation (fluorescent), second‐generation (phosphorescent), and third‐generation (TADF) emitters. F = fluorescence; phos = phosphorescence; PF = prompt fluorescence; DF = delayed fluorescence; ISC = intersystem crossing; RISC = reverse intersystem crossing; Δ*E*_ST_  = the energy gap between the first excited singlet and triplet states; nr = nonradiative. Reproduced with permission.^[^ ^1^ ^]^ Copyright 2020, Wiley.

TADF is often described as the endothermic transition of an exciton from the excited triplet state *T*_1_ into an excited singlet state *S*_1_ mediated by reverse intersystem crossing (RISC).^[^ ^6^ ^]^ It is worth mentioning that TADF materials are often referred to as the “third generation” OLED emitters, being the first and the second generations composed of purely fluorescent and phosphorescent emitters, respectively.^[^ ^1^ *^,^* ^7^ ^]^ As highlighted in **Figure** **1**, when electrons and holes are injected into the emissive layer of an OLED, they recombine to form 25% of singlet and 75% of triplet excitons according to spin statistics. The former quickly relaxes into prompt fluorescence (PF), while the latter repopulates the singlet state *S*_1_ through an endothermic process called “reversed intersystem crossing” (RISC), before being deactivated through delayed fluorescence (DF). Differently from purely organic TADF materials, the PF is sometimes not observable in TMCs due to the rapid ISC process.^[^ ^3^ *^,^* ^8^ ^]^

To observe TADF properties, the RISC process needs to fulfill the subsequent requirements. First of all, the energy gap Δ*E*_ST_ between the first excited singlet and triplet states (respectively *S*_1_ and *T*_1_) should be generally smaller than or equal to 1500 cm^−1^ (0.19 eV), although lower values are beneficial to achieve higher rate constants of the RISC process (*k*_RISC_).^[^ ^6^ ^]^ According to Boltzmann distribution, the *k*_RISC_ depends on Δ*E*_ST_, Boltzmann's constant *k*_B_, and the temperature T:

(1) $$k_{\mathrm{RISC}}\propto e^{\left(-\frac{\Delta E_{\mathrm{ST}}}{k_{B}T}\right)}$$

Consequently, *k*_RISC_ is increased at higher temperatures, although the PL quantum efficiency of the delayed fluorescence may decrease due to the activation of non‐radiative decay processes.^[^ ^4b^ ^]^ On the other hand, to achieve a small Δ*E*_ST_ the overlap of the highest occupied molecular orbital (HOMO) and the lowest unoccupied molecular orbital (LUMO) should be minimized, achievable via spatial separation, and leading to a small exchange interaction between the unpaired electrons.^[^ ^3^ ^]^ Therefore, short radiative TADF decay times, which are important to achieve high photoluminescent quantum yields (PLQYs), are expected. However, for compounds featuring an Δ*E*_ST_ below 200–300 cm^−1^, an increased *τ*(TADF) is detected.^[^ ^9^ ^]^ Moreover, the rate constant *k*_RISC_ should be much higher than *k*_ISC_ (ISC = intersystem crossing) and *k*_phos_ (phos = phosphorescence), and the triplet state *T*_1_ should be long‐lived since the combination with a high *k*_RISC_ determines an efficient repopulation of *S*_1_ that radiatively deactivates through DF.^[^ ^3^ ^]^

To qualitatively prove if a species exhibits TADF, the photoluminescence lifetime *τ* can be studied by lowering the temperature, typically from 300 K to 77 K when Δ*E*_ST_ is higher than 700 cm^−1^ (for smaller energy splittings, lower temperatures are required, for instance, *T* = 30 K). In a TADF material, *τ* increases since RISC is not thermally accessible and only *T*_1_ is effectively participating in the emission. On the other hand, at higher temperatures, the energetically higher *S*_1_ state is populated following Boltzmann distribution and determining a reduction in the observed *τ*. The emission at room temperature normally represents the TADF process together with a contribution from the *T*_1_ → *S*_1_ transition. Several parameters can be estimated based on the Arrhenius plot obtained considering the *τ* at different temperatures, i.e., *τ*(*S*_1_), *τ*(*T*_1_), and Δ*E*_ST_ (see Equation 2). It is worth mentioning that at very low temperatures the exponential terms of the equation can be neglected, and the measured *τ*(T) is equal to the phosphorescence lifetime *τ*(*T*_1_). On the other hand, at high temperatures and with long *τ*(*T*_1_), the term containing *τ*(*T*_1_) can be disregarded and *τ*(TADF) can be estimated.^[^ ^9^ ^]^

(2) $$\tau \left(T\right)=\frac{3+e^{-\frac{\Delta E_{\mathrm{ST}}}{k_{B}T}}}{\frac{3}{\tau \left(T_{1}\right)}+\frac{1}{\tau \left(S_{1}\right)}e^{-\frac{\Delta E_{\mathrm{ST}}}{k_{B}T}}}$$

**Figure 2:**
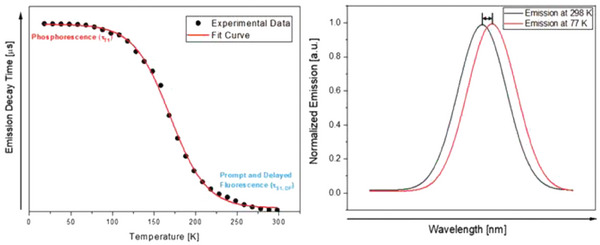
Plot of the emission decay times versus temperature highlighting respectively the phosphorescent and the TADF regions from low to ambient temperature (left); onset emission spectra at 298 K (black line) and 77 K (red line) highlighting the hypsochromic shift upon heating the sample (right). Reproduced with permission.^[^ ^3^ ^]^ Copyright 2020, Wiley.

As shown in **Figure** **2**, an additional aspect in evaluating if a material exhibits TADF is the bathochromic shift of the emission maxima on lowering the temperature, due to the emission originating from the lower‐lying triplet state.^[^ ^10^ ^]^ Nevertheless, while frequently observed, a bathochromic shift is not a *conditio sine qua non* for defining TADF systems, since in the solid matrix at low temperatures, the materials might experience different interaction effects with the matrix and/or change molecular packing.

Assuming that the total intensity *I_TOT_* =  *I*(*S*_1_) +  *I*(*T*_1_), where I(*S*_1_) and I(*T*_1_) are respectively the intensity originating from the first singlet and triplet states, and that the population of both states is governed by the Boltzmann distribution (i.e., fast equilibration), it is possible to determine the ratio between TADF and phosphorescence at room temperature:

(3) $$\;\frac{I\left(T_{1}\right)}{I_{TOT}}={\left[1+\frac{PLQY\left(S_{1}\right)\tau \left(T_{1}\right)}{3PLQY\left(T_{1}\right)\tau \left(S_{1}\right)}e^{-\frac{\Delta E_{\mathrm{ST}}}{k_{B}T}}\right]}^{-1}$$

Therefore:^[^ ^11^ ^]^

(4) $$\;\frac{I\left(S_{1}\right)}{I_{TOT}}=1-{\left[1+\frac{PLQY\left(S_{1}\right)\tau \left(T_{1}\right)}{3PLQY\left(T_{1}\right)\tau \left(S_{1}\right)}e^{-\frac{\Delta E_{\mathrm{ST}}}{k_{B}T}}\right]}^{-1}$$

To conclude, it is worth mentioning that DFT calculations can help to rationalize the experimental data, as well as the modeling of new TADF materials. However, this aspect is still challenging due to the nature of the process, especially when excited electronic states of different nature are involved, in combination with small energy gaps or even near degeneracies.^[^ ^3^ ^]^

## 3 OLEDs versus LECs

Electroluminescence is the physical phenomenon, where luminescence is produced by radiative recombination within a solid‐state material, i.e., the direct conversion of electric energy into light without heat dissipation. This optical process is the fundamental operational mode of light‐emitting diodes (LEDs), which are the ultimate general source of continuous light due to their high luminescence efficiency, quick response time, and long lifetime.

Since the first LED was built in the 1960s, using GaAs as electroluminescent inorganic crystals,^[^ ^12^ ^]^ the technology has evolved continuously, leading to energy‐efficient lighting solutions and diverse applications across various industries. The history of OLEDs dates to 1987 when the first practical OLED device was built using the green luminescent aluminum tris(8‐hydroxyquinolinate) (Alq_3_) as the emissive material.^[^ ^13^ ^]^

OLEDs offer several advantages over inorganic LEDs. For instance, they can be manufactured on large areas and flexible substrates, enabling the creation of bendable and rollable displays.^[^ ^14^ ^]^ Moreover, OLEDs are thin and lightweight, making them suitable for wearable applications.^[^ ^15^ ^]^ OLEDs can potentially be more energy‐efficient since they do not need the backlighting that is required in some inorganic LED configurations, they can display “true black” when all the pixels are switched off, and in some cases are even biodegradable, enabling an ecofriendly use of materials.^[^ ^16^ ^]^ Nevertheless, there are still numerous challenges ahead to advance in the field of OLEDs, such as the reduction of the driving voltage, the increase in the lifetime by lowering the degradation processes, and the improvement of the total efficiency.^[^ ^17^ ^]^

**Figure 3:**
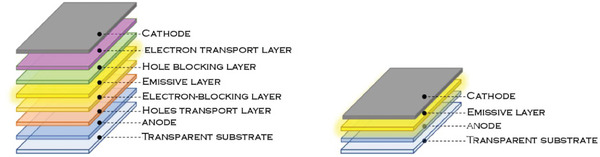
OLED (left) and LEC (right) architectures in comparison.

The basic structure of an OLED is constituted of multiple layers, sandwiched among a transparent anode, typically indium‐tin‐oxide (ITO), and a low work‐function metal cathode, such as Au or Al. When voltage is applied, electrons and holes are injected from the cathode and the anode, respectively, and migrate toward the emissive layer, forming excitons. To drive the charge carriers through the device with high mobility and avoid recombination outside of the emissive layer, the multilayer architecture has proven to be the most efficient (see **Figure** **3**, left).

In contrast, LECs have a quite appealing and straightforward design, since they are composed only of a single layer placed between the electrodes and characterized by mobile ionic molecules. The active layer in LECs is usually composed of a polymeric matrix, blended with a solid‐state electrolyte, or ionic transition metal complexes (iTMC).^[^ ^18^ ^]^ Upon application of a voltage, a strong interfacial electric field is generated, and the ionic species move toward the opposite‐charged electrode (see Figure 3, right). This architecture simplifies the operational process; thus, a LEC is insensitive to the work function of the cathode, allowing the employment of air‐stable metals, not requiring the matching of the Fermi energy with the HOMO‐LUMO energy levels of the electroluminescent material, and making it able to work upon a reversed applied bias. Moreover, the manufacturing of a LEC can be achieved through simple solution‐process techniques, while OLEDs sometimes require vacuum‐deposition approaches. Thus, LEC technology has been primarily exploited in academic research, where novel emitters or other materials could be directly tested, also because their worse performances in terms of brightness and efficiency currently limit their possible industrial application.^[^ ^19^ ^]^

The performance of OLED and LEC devices can be estimated considering the external quantum efficiency (EQE), which is defined as the ratio of photons extracted against the injected charges, and it is the product of the internal quantum efficiency (IQE) and the outcoupling constant (η_out_), as highlighted in Equation 5. IQE depends on the charge balance factor (γ), the ratio of excitons that contribute to the emission (η_S/T_), and the PLQY, as the ratio between emitted and absorbed photons.^[^ ^4b^ ^]^

(5) $$\mathrm{EQE}=\mathrm{IQE}\cdot \eta_{\mathrm{out}}=\gamma \cdot \eta_{S/T}\cdot \mathrm{PLQY}\cdot \eta_{\mathrm{out}}$$

γ is ideally equal to 1, as it indicates whether equality between the injected holes and electrons is observed, and the ratio of exciton formation by recombination. As previously highlighted, upon electrical excitation, present in both OLEDs and LECs, 25% singlet and 75% triplet excitons are formed according to spin statistics. Consequently, the harvesting of both singlet and triplet states is required to achieve IQE = 1, and therefore the luminescent material should be either phosphorescent or exhibit TADF.^[^ ^20^ ^]^

## 4 Earth‐abundant TMCs

### 4.1 Group 4: Zr(IV) Complexes

**Figure 4:**
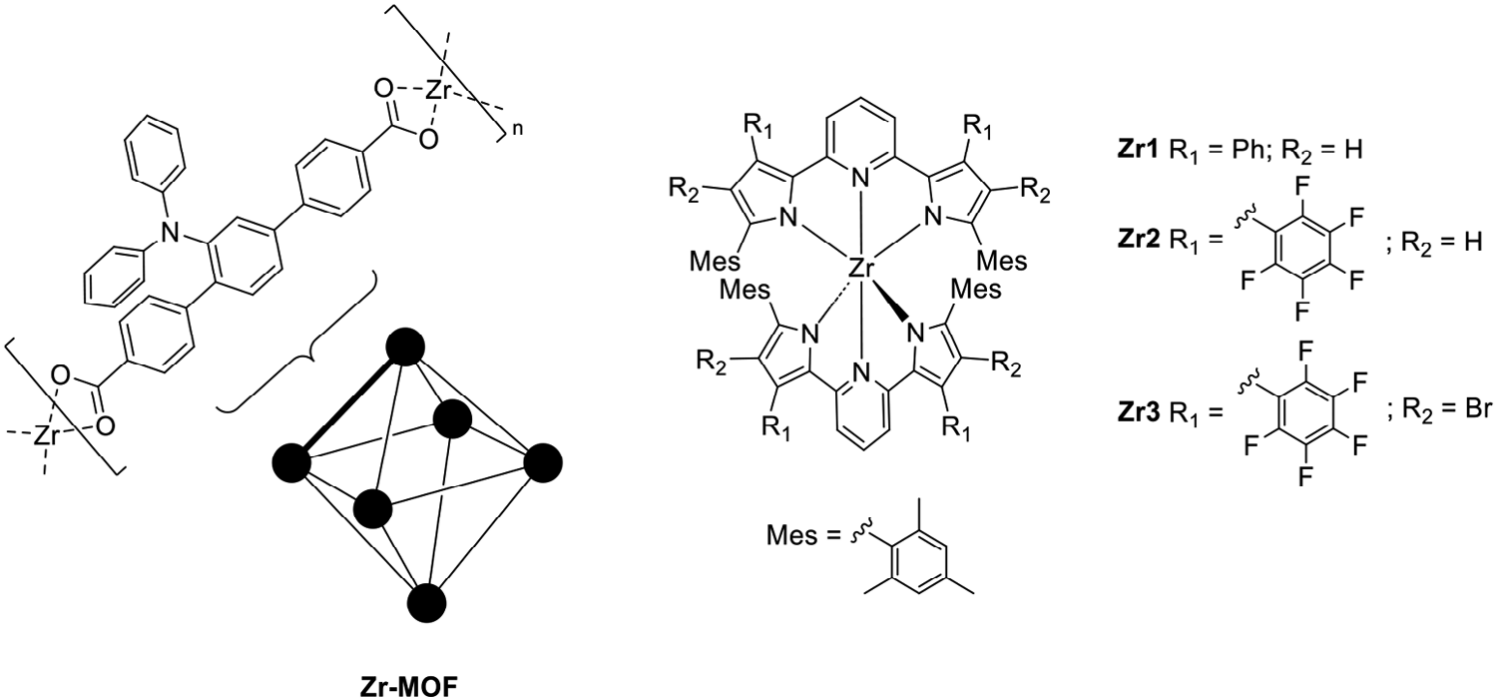
Structures of Zr‐MOF and other hexacoordinated Zr(IV) complexes exhibiting TADF properties.

Zirconium is present in the earth's crust at concentrations ≈165 ppm, and TADF properties were first observed in a Zr(IV)‐based metal‐organic framework (MOF) designed by Adachi, Kabe, and coworkers. In the described MOF structure, a terphenyl derivative acts as an organic linker, bearing two carboxylic groups as the electron‐accepting and Zr‐coordinating moieties, and a diphenylamine as the electron‐donating unit (see Zr‐MOF, **Figure** **4**). Therefore, the donor and acceptor moieties result in a confined localization of HOMO and LUMO onto different entities of the luminescent ligand, leading to a calculated ΔE_ST_ of 1613 cm^−1^ (0.20 eV). When the zirconium is coordinated in the MOF structure (UiO‐68), the emission is shifted from 461 to 501 nm in the solid state. The PLQY is reduced from 32% to 18%, with a *τ*(PF) of 17 ns and a *τ*(DF) of 0.18 ms, ≈1000 times faster than the pure organic linker. The delayed emission intensity increases with the temperature, which is characteristic of TADF behavior. Further effects of the Zr(IV) ion were not explicitly investigated.^[^ ^21^ ^]^

**Figure 5:**
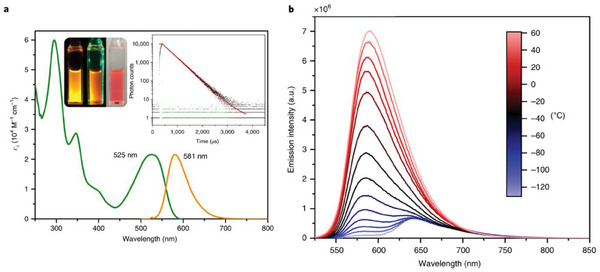
a) Absorption and emission spectra of **Zr1** in THF under nitrogen atmosphere; inset of the THF solution under *λ*_ex_ = 254 nm, 530 nm and ambient light irradiation, and photoluminescent decay lifetimes. b) Temperature‐dependent emission spectrum in 2‐methyl THF at different temperatures. Reproduced with permission.^[^ ^22^ ^]^ Copyright 2020, Springer Nature.

Milsmann and coworkers designed a Zr(IV) complex exhibiting TADF characteristics. Since the metal center is electron deficient, having a 4d^0^ configuration, the excited state has a ligand‐to‐metal charge‐transfer (LMCT) character. Thus, the design of electron‐rich ligands is essential for this complex. The strategy used by the authors was to prepare derivatives of the tridentate pyridinedipyrrolide (PDP) ligand. In particular, complex **Zr1** showed TADF at room temperature, presenting a structureless profile, with a *λ*_max_ centered at 581 nm (see **Figure** **5**, left). The PLQY and the excited state lifetimes in solution are 45% and 350 ms, respectively. The nature of the excited state was assigned to a mixed IL/LMCT character based on TD‐DFT calculations. As observable in Figure 5 (right), the emission spectra collected at different temperatures showed a second band at 642 nm below 253 K, which is the sole emission band at 143 K. The temperature dependence of the emission profile was additionally evaluated according to the PL rate constant, suggesting that the *S*_1_ and *T*_1_ states of **Zr1** are in thermal equilibrium with an energy gap of 1653 cm^−1^ (0.20 eV). The rigidochromism prevails at lower temperatures; thus, at 77 K, the emission profile becomes more structured with two main peaks at 620 and 670 nm. This compound was used as a photosensitizer in different photoredox reactions, undergoing either oxidative or reductive quenching, depending on the presence of electron acceptor or donors.^[^ ^22^ ^]^ A few years later, the same authors successfully employed **Zr1** in photodynamic therapy.^[^ ^23^ ^]^

Russegger et al. reported a series of photostable TADF emitting Zr(IV) complexes with a modified design of the PDP ligands (see **Zr2‐Zr3** as selected examples in Figure 4). They used differently substituted phenyl derivatives, introducing electron‐donor or acceptor groups in addition to halides in the 4‐position of the pyrrole moiety. The Zr(IV) derivatives emit in the orange‐red region of the visible spectrum, with maximum wavelengths ranging from 589 to 600 nm in toluene. Interestingly, their emission is attributed to delayed fluorescence (DF) emission only, without any contribution from prompt fluorescence (PF) and with PLQYs up to 50% in solution. The presence of electron‐withdrawing or donating groups does not have a large effect on the emission maxima and PLQYs. On the other hand, the excited state lifetimes *τ* are strongly affected by the different substituents, in particular the groups on the phenyl ring, and by the presence of the halides. Compared to **Zr1**, the *τ*(TADF) decreases from 282 to 194 µs in the case of **Zr2** and to 96 µs with the bromo‐species **Zr3**, which can be justified considering the heavy atom effect and thus the larger SOC. A similar trend could be observed when the emitters were dispersed in a polystyrene film. Although the emission shape and position do not change, the rigid environment increases the PLQY up to 100%. As the compounds were designed as optical temperature sensors, analyses of the dependence of the lifetimes according to temperature were performed between 283 and 323 K under anoxic conditions. The authors observed an exponential decrease of the TADF decay times for all the Zr(IV) complexes, with a sensitivity between −2.5 and 2.9% per Kelvin, implying an excellent performance as luminescent sensors.^[^ ^24^ ^]^

### 4.2 Group 6: W(VI) Complexes

**Figure 6:**
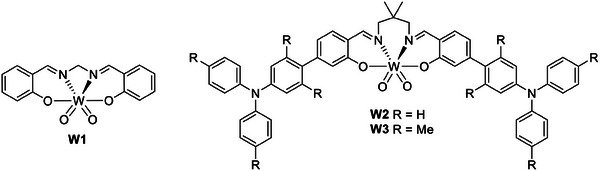
Chemical structures of the W(VI) complexes exhibiting TADF and employed in the fabrication of OLEDs.

A less abundant, but still cost‐effective and interesting metal is tungsten, which is distributed in the earth's crust with 1 ppm concentration. The design of TADF compounds based on this metal is quite appealing, also given their air stability and SOC constant comparable to that of Ir and Pt (*ξ*_W_ = 2443 cm^−1^ vs. *ξ*_Ir_ = 3909 cm^−1^ and *ξ*_Pt_ = 4481 cm^−1^). As with Zr(IV), W(VI)‐based complexes have a d^0^ electronic configuration but, in this case, the emission is ascribed to an ILCT process. Ligands employed in the coordination of W(VI) are often Schiff bases, such as those depicted in **Figure** **6**. The ligands are mainly responsible for the absorption bands, assigned to ligand‐centered transitions, while the lowest absorption band is assigned to an intraligand charge transfer excited state with contribution from a LMCT.

The first OLED containing the emissive W(VI) complex W1 exhibiting TADF was prepared by Che and coworkers in 2017. Photophysical investigations revealed that W1 emits at 600 nm with a PLQY of 2.8% in dichloromethane. The PLQY increases about ten‐fold in a 5 wt.%‐doped 1,3‐bis(*N*‐carbazolyl)benzene (mCP) film. Although this emission was assigned to a ^3^IL transition, based on TD‐DFT calculations, the Δ*E*_ST_ is estimated at ≈573 cm^−1^ (0.07 eV), suggesting TADF behavior. A prototypical OLED was fabricated with the following structure: ITO/PEDOT:PSS/PYD2:**W1**(4%)/DPEPO/TPBi/LiF/Al (ITO = indium tin oxide; PEDOT:PSS = poly(3,4‐ethylenedioxythiophene):polystyrene sulfonate; PYD2 = 2,6‐dicarbazolo‐1,5‐pyridine; DPEPO = bis[(2‐diphenylphosphino)phenyl] ether dioxide; TPBi = 2,2′,2″‐(1,3,5‐benzinetriyl)‐tris(1‐phenyl‐1H‐benzimidazole)). An EQE_max_ of 4.79% was obtained at a luminance of 40 cd m^−2^.^[^ ^25^ ^]^

In 2019, Chan et al. improved the design by introducing electron donor arylamino groups, which caused a large change in the dipole moment together upon excitation. W2 emits in the yellow region, with a broad and structureless emission centered at 554 nm and 56% PLQY in the toluene solution. The quantum yield increased up to 84% when it was used as a dopant in a mCP film.^[^ ^26^ ^]^ Interestingly, **W2** was efficiently used as a photocatalyst in several photoinduced reactions, such as borylation of aryl halides and homocoupling of benzylic halides.^[^ ^27^ ^]^ The lower performances of **W3** (*λ*_max_ = 567 nm, 49% PLQY and *τ* = 228 µs, 5 wt.% in mCP) were attributed to the presence of the methyl groups on the arylamino moiety that forced a spatial separation between the HOMO and LUMO, respectively localized on the arylamino moiety and the Schiff base. The calculated charge density difference supported the ILCT nature of the excited state and the Δ*E*_ST_ was estimated at ≈2795 cm^−1^ (0.35 eV) for **W2** and 748 cm^−1^ (0.09 eV) for **W3**. Therefore, the emission was ascribed to pure phosphorescence in the former case, and TADF in the latter. An additional comparison was given by the fabrication of the corresponding solution‐processed OLEDs. The devices were composed by an ITO/PEDOT:PSS/PVK:OXD‐7:W(VI)‐emitter:TPBi/LiF/Al, where OXD‐7 is 1,3‐bis(4‐tert‐butyl‐phenyl)−1,3,4‐oxadiazolyl)phenylene. The electroluminescent spectra of both the OLEDs are characterized by almost the same maximum wavelength (circa 580 nm). Owing to the short emission lifetime, the device employing **W3** as the emitter performed better than the one containing **W2**. The maximum EQE achieved was 15.6% with 30 wt.% of **W3** in the PVK film.^[^ ^26^ ^]^

### 4.3 Group 11: Cu(I) Complexes

After the pioneering work of McMillin and coworkers,^[^ ^28^ ^]^ Cu(I) complexes received growing attention to be employed as emitters in optoelectronics. Besides being an earth‐abundant element, one of the major advantages of Cu(I) derivatives is the possibility of tuning the absorption and emission properties by modifying the coordinated ligands.^[^ ^29^ ^]^

**Figure 7:**
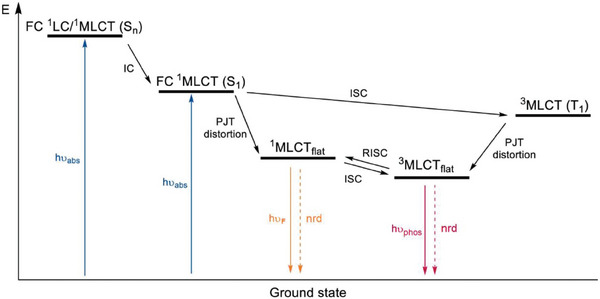
General scheme for emissive tetracoordinated Cu(I) complexes. Abbreviations: internal conversion (IC), intersystem crossing (ISC), Franck‐Condon (FC), metal to ligand charge transfer (MLCT), nonradiative deactivation (nrd), pseudo‐Jahn–Teller distortion (PJT distortion), reverse intersystem crossing (RISC), absorption (abs), fluorescence (F), flattened geometry (flat), phosphorescence (phos). Reproduced with permission.^[^ ^30a^ ^]^ Copyright 2022, American Chemical Society.

Cu(I) has a d^10^ configuration and its complexes are typically two‐, three‐ or four‐coordinated. In the latter case, the geometry is tetrahedral or distorted tetrahedral to minimize steric repulsions. The completely filled *d*‐shell determines a symmetrical electronic distribution and prevents *d*‐*d* metal‐centered transitions that can quench the emission. As highlighted in **Figure** **7**, in most cases, the mechanism responsible for the emission is related to a metal‐to‐ligand charge transfer (MLCT) process where the metal is formally oxidized to Cu(II) with consequent Jahn‐Teller distortion to the square planar geometry. This deformation facilitates non‐radiative decay paths that quench the emission but can be avoided by introducing sterically hindered ligands. At the excited state, the Cu(II) center may coordinate a new ligand (e.g., solvent molecules or counter ions) to fulfill an available site in the perpendicular position of the square planar complex, with the formation of a five‐coordinated “exciplex” that undergoes non‐radiative deactivation.^[^ ^29^ ^]^

The possibility of harvesting both singlet and triplet excitons through TADF makes Cu(I) complexes suitable candidates to be applied in OLEDs and LECs.^[^ ^8^ *^,^* ^30^ ^]^ Being a first‐row transition metal, the SOC is much more limited in Cu(I) (*ξ*_Cu_ = 857 cm^−1^) compared to Ir(III) and Pt(II) derivatives, but the rate constants *k*_ISC_, *k*_RISC_, and *k*_phos_ are sufficiently high to determine an equilibrium between *S*_1_ and *T*_1_ which is governed by the Boltzmann distribution.^[^ ^9^ *^,^* ^31^ ^]^ In contrast to purely organic compounds, in Cu(I) derivatives the phosphorescent process from *T*_1_ competes from a kinetic point of view with the non‐radiative decay from the triplet state (*k* ^T^_nr_) and *k*_RISC_.^[^ ^1^ ^]^

In the following pages, the Cu(I) complexes will be divided according to the ligands employed. However, it is worth mentioning that some papers that investigated heteroleptic Cu(I) complexes were published before the importance of TADF was fully understood so the number of derivatives exhibiting this feature may be much bigger than the examples discussed here.

#### 4.3.1 N^P and N^As ligands

Cu(I) complexes bearing N^P ligands represent a wide family of derivatives due to the variety of architectures that can be obtained by employing different Cu(I) precursor stoichiometries and the possible coordination through both the phosphorus and the nitrogen atoms.^[^ ^32^ ^]^

**Figure 8:**
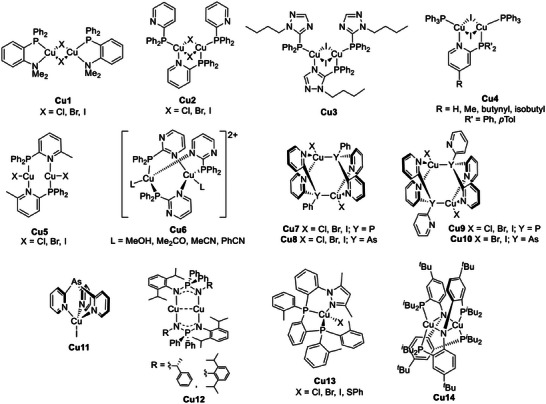
Cu(I) complexes having N^P ligands and TADF properties.

The first ligands investigated by Yersin and coworkers were chelating aminophosphanes, such as 2‐(diphenylphosphaneyl)‐*N*,*N*‐dimethylaniline, in combination with CuX (X = Cl, Br, I) as precursor. The corresponding binuclear Cu(I) complexes **Cu1** depicted in **Figure** **8** were characterized by blue and green TADF emission ascribed to (M+X)LCT. At 300 K, the bromo derivative showed a PLQY of 65% and *τ*(DF) equal to 4.1 µs, and the emission was found to originate from the S_1_ → *S*_0_ transition (98%). At 77 K, the lifetimes were in the hundreds of microseconds range, and the Δ*E*_ST_ was estimated ≈ at 500 cm^−1^ (0.06 eV).^[^ ^11^ ^]^

**Figure 9:**
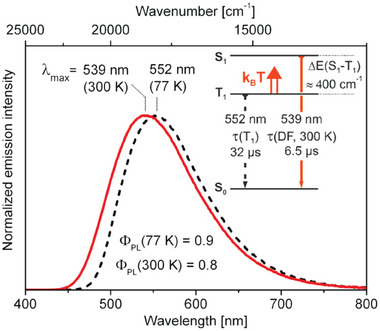
Emission spectra, PLQY, lifetimes t, and energy diagram for [Cu_2_(µ‐I)_2_(PyrPhos)_3_] as inset. Reproduced with permission.^[^ ^33^ ^]^ Copyright 2013, American Chemical Society.

Another N^P ligand studied by Yersin and coworkers was 2‐(diphenylphosphino)pyridine (PyrPhos) and the corresponding dinuclear Cu(I) complexes **Cu2** (see Figure 8) exhibited green emissions. As highlighted in **Figure** **9**, for [Cu_2_(µ‐I)_2_(PyrPhos)_3_] at 300 K the emission maximum was centered at 539 nm with *τ*(DF) = 6.5 µs. At 77 K, the emission peaked at 552 nm and the lifetime *τ*(*T*_1_) was equal to 32 µs. DFT calculations highlighted that the charge transfer process involves the HOMO localized on the Cu_2_(µ‐X)_2_ cluster and the LUMO confined on the bridging PyrPhos ligand.^[^ ^33^ ^]^ Similar photophysical properties were detected substituting the pyridine fragment with pyrazine or isoquinoline.^[^ ^34^ ^]^ The introduction of an *N*‐*n*‐butyl‐substituted 1,2,4‐triazole (see **Cu3**, Figure 8) determined the separation of the zero‐field splitting (ZFS) of the first triplet state *T*_1_ into substates divided by ≈2.5 cm^−1^ (0.3 meV). Below 10 K, spin‐lattice relaxation is also observed and in line with the ZFS.^[^ ^35^ ^]^

When triphenylphosphine was employed as an ancillary ligand, luminescent butterfly‐shaped Cu(I) complexes **Cu4** having the general formula [Cu_2_(µ‐I)_2_(PyrPhos)(PPh_3_)_2_] were isolated (see Figure 8). Based on experimental data and DFT calculations, the derivatives exhibited green to yellow emissions that were ascribed to TADF. The introduction of substituents in the *para*‐position of PPh_3_ did not modify the photophysical properties of the corresponding Cu(I) complexes but did affect their solubility, in particular when tris(*p*‐fluorophenyl)phosphine was used.^[^ ^36^ ^]^ This aspect is particularly relevant for the preparation of solution‐processed OLEDs. The Cu(I) species **Cu4** having 2‐(bis(4‐methylphenyl)phosphino)−4‐methylpyridine as N^P ligand were efficiently employed for such an application, and the corresponding devices exhibited brightness of 5900 cd m^−2^ and current efficiency of 3.79 cd A^−1^.^[^ ^37^ ^]^

**Figure 10:**
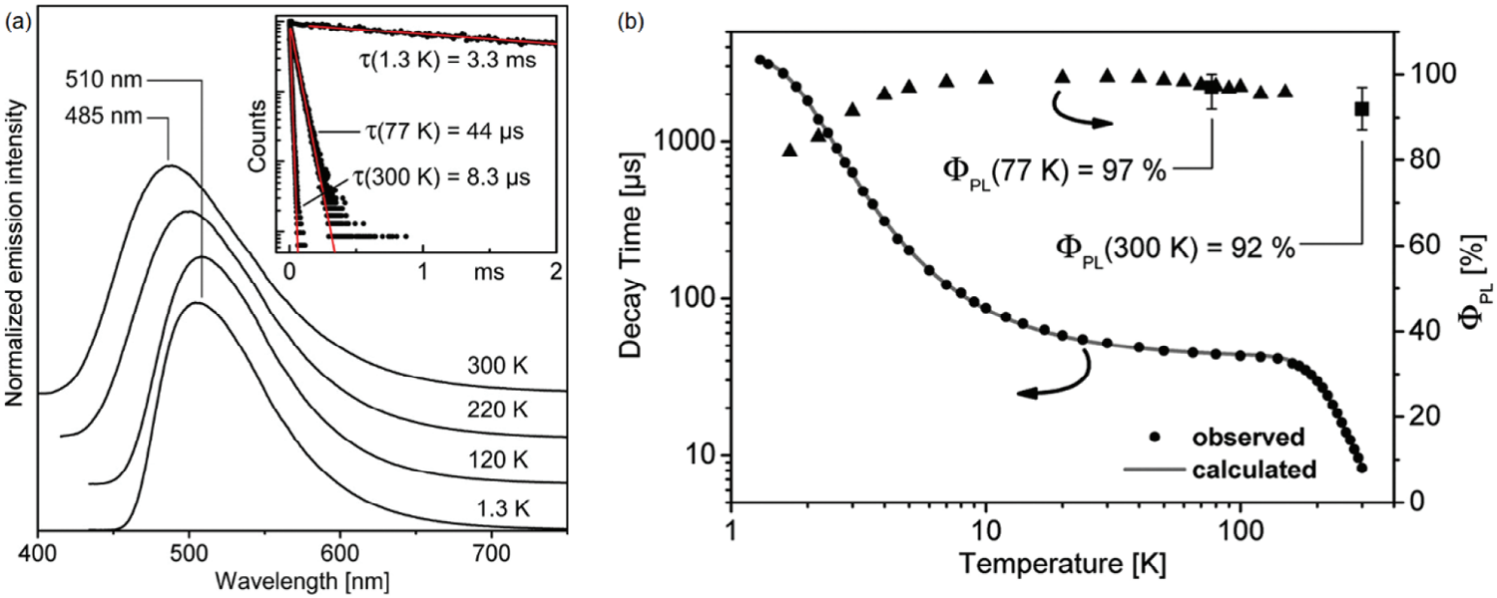
a) Emission spectra of [Cu_2_Cl_2_(MePyrPhos)_2_] at 1.3, 120, 220, and 300 K. Inset: photoluminescent lifetimes at 1.3, 77, and 300 K. b) Photoluminescent lifetimes and PLQY at variable temperature, fitted according to Equation (2). Reproduced with permission.^[^ ^38^ ^]^ Copyright 2015, American Chemical Society.

Similar photophysical properties were detected when 2‐(diphenylphosphino)−6‐methylpyridine (MePyrPhos) was employed as a ligand and reacted with CuX (X = Cl, Br, I) in the ratio 1:1 (see **Cu5** in Figure 8). The blue‐emitting [Cu_2_Cl_2_(MePyrPhos)_2_] was characterized by a PLQY of 92% at 300 K, which increased to 97% at 77 K. As observable in **Figure** **10**, the emission maximum was located at 510 nm between 1.3 K and 120 K, whereas it was blue‐shifted to 485 nm at higher temperatures. Passing from 1.3 K to 77 K the photoluminescence lifetime was drastically reduced from 3.3 ms to 44 µs, while it was ≈8 µs at room temperature. The Δ*E*_ST_ was estimated ≈1000 cm^−1^ (0.12 eV), and owing to the large ZFS equal to 15 cm^−1^ and the high *k*_phos_ of 2.4 × 10^4^ s^−1^, at room temperature the emission was composed by two contributions, direct phosphorescence (20%) and TADF (80%).^[^ ^38^ ^]^

The reaction between [Cu(NCCH_3_)_4_]^+^ and diphenyl(2‐pyrimidyl)phosphine (PymPhos) in the ratio 3:2 determined the formation of paddle‐wheel dinuclear Cu(I) complexes **Cu6** (see Figure 8). The coordination sphere around the metal center was completed by different solvent molecules, i.e., methanol, acetone, acetonitrile, or benzonitrile. Based on variable temperature photoluminescence and DFT calculations, the room‐temperature TADF emission was ascribed to a ^1^(M+PyrPhos+L)LCT, with the ancillary ligands influencing the emission maxima (*λ*_max_ = 500–563 nm) and resulting in a Δ*E*_ST_ in the range 550–1100 cm^−1^ (0.07‐0.14 eV). At 77 K the phosphorescence had the same ^3^(M+PyrPhos+L)LCT character.^[^ ^39^ ^]^ In combination with 1,4‐dicyanobenzene (DCB), the same group reported also the 1D coordination polymer [Cu_2_(PymPhos)_3_(DCB)_n_]^2+^ and the dinuclear Cu(I) complex [Cu_2_(PymPhos)_6_(DCB)]^2+^, both exhibiting TADF properties. Unlike the Cu(I) derivatives **Cu6** previously described, in the latter binuclear Cu(I) complex, PymPhos does not act as an N^P ligand, but it is coordinated to the metal center only through the phosphorus atom.^[^ ^40^ ^]^

Substituting the phosphorus atom with another pnictogen element, such as arsenic, leads to a significantly shorter photoluminescent lifetime at room temperature. Artem'ev and coworkers described the use of bis(2‐pyridyl)phenyl arsine (py_2_AsPh) as a tridentate ligand for the Cu(I) complexes **Cu8** (see Figure 8). The measured lifetime was between 2 and 9 µs at 300 K, while for the analogs **Cu7**, it was in the 5–33 µs range.^[^ ^41^ ^]^ This outcome was rationalized considering the larger Δ*E*_ST_ observed for the arsine Cu(I) complexes that determined a mixing between TADF and fast phosphorescence at room temperature. Another aspect to be mentioned is the larger SOC effect originated by arsenic compared to phosphorus (*ξ*_As_ = 1202 cm^−1^ vs. *ξ*_P_ = 230 cm^−1^) that increases the radiative decay constant *k*_r_. At 300 K, the ratio between TADF and phosphorescence increased passing from Cl to Br and I. The halide directly influences the Δ*E*_ST_ which favors TADF instead of phosphorescence.^[^ ^41b^ *^,^* ^42^ ^]^ Similar dual photoluminescent properties were detected employing tris(2‐pyridyl)phosphine (py_3_P) and tris(2‐pyridyl)arsine (py_3_As) as tridentate ligands in combination with Cu(I) halides (see **Cu9** and **Cu10** in Figure 8). In the case of the iodo‐derivative and tris(2‐pyridyl)arsine (py_3_As), the complex **Cu11** where the ligand acted as scorpionate was also isolated, and the Cu(I) species exhibited TADF properties due to (M+X)LCT.^[^ ^42^, ^43^ ^]^

Roesky and coworkers reported using chiral iminophosphonamides as N^P^N ligands to afford blue‐emitting Cu(I) complexes **Cu12** exhibiting TADF (see Figure 8). The geometry around the metal center is almost linear with the two Cu(I) centers at close distances thus enabling metallophilic interactions. In contrast to the previously described Cu(I) derivatives, for **Cu12** the emission was almost absent. For T > 150 K the overall emission was negligible, attributed to non‐radiative (internal) conversion from *S*_1_ to *S*_0_, with *S*_1_ being populated by RISC from *T*_1_. In analogy to TADF, the authors labeled the process as “thermally activated phosphorescence quenching”.^[^ ^44^ ^]^

To conclude, similarly to the N^P^N ligands previously presented, N^P^P and P^N^P tridentate ligands were employed to prepare Cu(I) complexes characterized by TADF properties. In the former case, the corresponding neutral Cu(I) derivatives **Cu13** having Cl, Br, I, or SPh in the coordination sphere were successfully isolated and characterized by Yersin et al. (see Figure 8). The Cu(I) complexes **Cu13** exhibited an overall yellow‐green emission with *τ* in the µs range at 300 K and up to 3.3 ms at 77 K. For all the derivatives the PLQY was between 70 and 90% at both temperatures, and the TADF decay rate *k*_r_ was estimated between 9.2 × 10^4^ and 18 × 10^4^ s^−1^. These values were two orders of magnitude lower at 77 K. Due to the promising photoluminescent features of the iodo‐ and the phenylthiolato‐species, these complexes were tested to prepare OLEDs containing 2, 4, and 8 wt.% of the emitter. The highest EQE (16.4%) was detected for the Cu(I) iodo‐derivative (2 wt.%), which achieved luminance of ≈10000 cd m^−2^.^[^ ^45^ ^]^ A device with similar performances was previously prepared using the homoleptic binuclear Cu(I) complex **Cu14** obtained using bis(2‐diisobutylphosphinophenyl)amide) as tridentate P^N^P ligand (see Figure 8).^[^ ^46^ ^]^

#### 4.3.2 N^N Ligands

**Figure 11:**
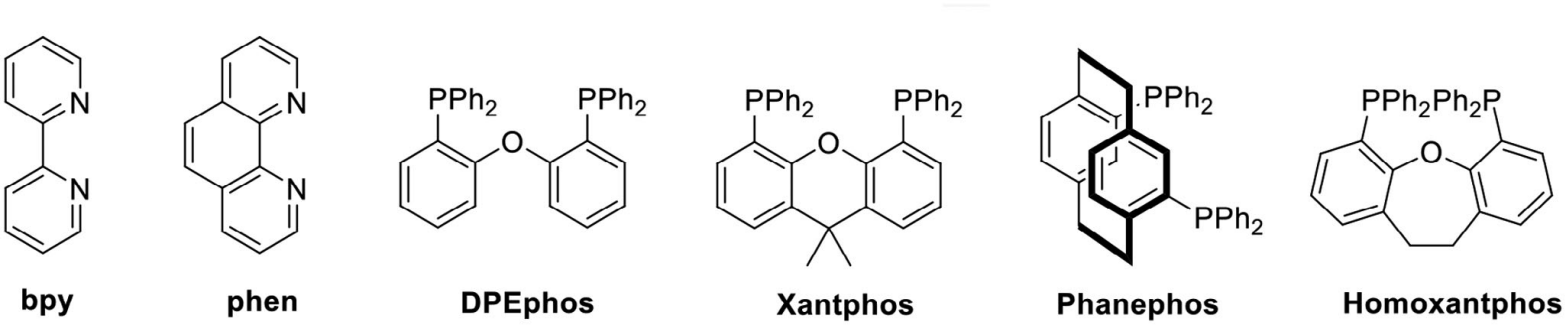
N^N and P^P ligands are commonly employed in heteroleptic Cu(I) complexes.

Another family of ligands for Cu(I) complexes is composed of N^N ligands such as 2,2′‐bipyridine (bpy) and 1,10‐phenanthroline (phen), depicted in **Figure** **11**. Differing from homoleptic derivatives, heteroleptic Cu(I) complexes having the general formula [Cu(N^N)(P^P)]^+^ (P^P = phosphine) are characterized by improved photoluminescent properties due to the decreased non‐radiative deactivation compared to [Cu(N^N)_2_]^+^.^[^ ^47^ ^]^ It is worth noting that in this case, the HOMO is normally a *σ*‐bonding orbital involving the Cu(I) metal center and the phosphines, while the LUMO is localized on the N^N ligands.^[^ ^6^ ^]^ Since both N^N and P^P ligands participate directly in the emission mechanism, even small changes in their skeleton, for instance introducing aromatic substituents in the 2,9 positions of phen,^[^ ^48^ ^]^ can induce large differences in the photoluminescent features in the resulting Cu(I) complexes.^[^ ^29a^ ^]^ Bis[(2‐diphenylphosphino)phenyl] ether (DPEphos) and (9,9‐dimethyl‐9H‐xanthene‐4,5‐diyl)bis(diphenylphosphine) (Xantphos) are often employed as P^P ligands owing to their wide‐bite angles and rigid structure that prevents the pseudo‐Jahn‐Teller distortion at the excited state (see Figure 11).^[^ ^49^ ^]^ In addition, it was observed that π‐π stacking interactions between the phenyl rings of DPEphos and the N^N ligands (e.g., phen) can further enhance the emissive properties, in particular the PLQY.^[^ ^50^ ^]^

**Figure 12:**
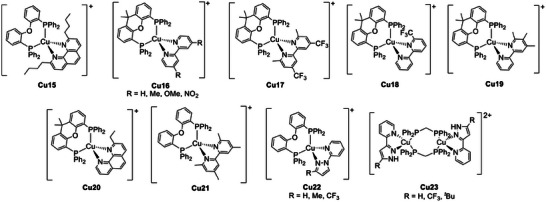
Cationic Cu(I) complexes exhibiting TADF.

As previously stated, [Cu(phen)(PPh_3_)_2_]^+^ was the first Cu(I) complex exhibiting TADF properties, passing from a greenish delayed fluorescence at 300 K to a yellow phosphorescence once cooled down at 4.2 K.^[^ ^5^ *^,^* ^28^ ^]^ The introduction of substituents in the 2,9‐positions of the phen ligand and the use of previously reported bidentate phosphines determined a wide library of Cu(I) complexes characterized by high PLQYs.^[^ ^48^ ^]^ In this category, the first Cu(I) derivative to be applied for OLED technology was **Cu15** (see **Figure** **12**), and the corresponding device ITO/PEDOT:PSS/16 wt.% Cu(I) complex: PVK/BCP/Alq_3_/LiF/Al (ITO as anode; PEDOT:PSS as hole injection and transport layer; PVK = polyvinylcarbazole as host material; BCP = 2,9‐dimethyl‐4,7‐diphenyl‐1,10‐phenanthroline as triplet exciton blocking and electron transport layer; Alq_3_ as electron injection layer; LiF as buffer layer; Al as cathode) exhibited promising performance both in terms of current efficiency (10.5 cd A^−1^ at 105 cd m^−2^) and brightness (1663 cd m^−2^).^[^ ^4b^ *^,^* ^51^ ^]^ The same complex was employed for the preparation of a LEC device characterized by a green emission centered at 523 nm, current efficiency up to 56 cd A^−1^ at 4 V and 16% external quantum efficiency (EQE), suggesting that both singlet and triplet excitons were involved in the emissive mechanism.^[^ ^52^ ^]^

**Figure 13:**
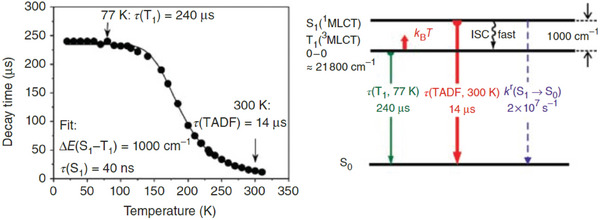
a) Emission lifetimes *τ* (µs) versus temperature (K) measured for [Cu(dmp)(phanephos)]^+^ (solid sample, *λ*_ex_ = 355 nm, *λ*_em_ = 550 nm). b) Energy level diagram. Reproduced with permission.^[^ ^9^ ^]^ Copyright 2017, Wiley.

Moreover, the TADF properties were improved by Czerwieniec and coworkers employing the rigid 4,12‐bis(diphenylphosphine)‐[2,2]paracyclophane (phanephos, Figure 11) as P^P ligand in combination with 2,9‐dimethyl‐1,10‐phenantroline (dmp) as N^N. The corresponding Cu(I) complex exhibited a yellow‐green emission centered at 530 nm with 80% PLQY and 14 µs lifetime at room temperature (*k*_r_ = 6 × 10^4^ s^−1^). At 77 K the emission maximum was red‐shifted to 562 nm and *τ* increased to 240 µs (the corresponding radiative decay *k*_r_ decreased to 3 × 10^3^ s^−1^). As highlighted in **Figure** **13**, this value did not change between 120 K and 20 K, and it was assigned to the *T*_1_ → *S*_0_ transition. On the other hand, the lifetime measured at room temperature was associated with the TADF process, and *τ*(PF) was estimated at ≈40 ns.^[^ ^53^ ^]^

Zysman‐Colman and coworkers investigated the role of the chelating diphosphine in complexes having the general formula [Cu(dmp)(P^P)]^+^, noticing that in the solid state, the PLQY is directly influenced by the percent volume buried (%V_bur_). The derivative having 10,11‐dihydro‐4,5,‐bis(diphenylphosphino)dibenzo[*b*,*f*]oxepine (homoxantphos, Figure 11) as chelating P^P showed the highest %V_bur_ and PLQY, which impacted the external quantum efficiency of the derived LEC (EQE = 1.9%) and OLED (EQE = 4.4%). The LEC device had a much longer lifetime compared to the previously described **Cu15** (1.25 vs. over 16.5 h) and exhibited the best performances due to the stability of both oxidized and reduced species.^[^ ^54^ ^]^

Another parameter that influences the photo‐ and electroluminescence features (i.e., PLQY, *τ*, emission maxima, device brightness, stability, and efficacy) is the *σ*‐Hammett parameter (*σ*_p_), i.e., the *σ*‐donation effect when employing *para*‐substituted bpy as N^N. Methoxy (*σ*_p_ = 0.27), methyl (*σ*_p_ = 0.17), and nitro groups (*σ*_p_ = 0.78) were compared to the unsubstituted bpy (*σ*_p_ = 0) in the heteroleptic Cu(I) complexes **Cu16** (see Figure 12). Except when bpy^NO2^ was used as N^N, **Cu16** exhibited TADF properties and was employed to prepare LECs. The derived devices consisted of a 90 nm active layer containing the complex, which was placed between an ITO bottom electrode covered with a thin PEDOT:PSS layer and an evaporated aluminum top electrode. **Cu16** having bpy^OMe^ as a chelating ligand exhibited the best performances, resulting in an overall five‐fold enhancement in terms of luminance (50 cd m^−2^), efficiency (0.35 cd A^−1^) and stability (1000 mJ cm^−2^) compared to the LEC obtained with the unsubstituted bpy Cu(I) derivative.^[^ ^55^ ^]^

To shorten the time required to achieve the maximum luminance also known as “turn‐on lifetime” (t_on_), Housecroft and coworkers introduced 1‐ethyl‐3‐methylimidazolium hexafluorophosphate [Emim][PF_6_], causing an increased concentration of ionic species which boosted the mobility in the light‐emitting layer. Consequently, the LEC utilizing Cu17 had the fastest turn‐on time (8 min.), while the LEC using **Cu18** had the longest lifetime (*t*_1/2_ = 31 h). The LECs fabricated from Cu17 and Cu18 had a maximum luminance of 131 and 109 cd m^−2^, respectively.^[^ ^56^ ^]^ The same authors recently further improved the LEC performance by employing alkyl‐substituted bpy and phen, achieving maximum luminance up to 462 cd m^−2^ and t_1/2_ of 98 h when **Cu19** and **Cu20** were employed as emitters.^[^ ^57^ ^]^

A similar Cu(I) complex, having 4,4′,6,6′‐(tetramethyl‐2,2′‐bipyridyl) and DPEphos, respectively as N^N and P^P ligands (see **Cu21**, Figure 12), was the first example of TADF derivative which was reported to be active toward free radical and cationic photopolymerizations. The photopolymerization reactions were carried out under air employing a violet LED centered at 405 nm, and the Cu(I) complex was introduced in two‐ or three‐component systems in combination with an iodonium salt or amine, *N*‐vinyl carbazole or 9*H*‐carbazole‐9‐ethanol (CARET). The photocatalytic activity was compared with a non‐TADF Cu(I) complex having 4,4′‐dimethyl‐2,2′‐bypiridyl as N^N ligand, highlighting the beneficial effect of TADF materials on the photopolymerization process, particularly on the conversion. At the same time, **Cu21** exhibited enhanced photocatalytic performances compared to pure organic TADF molecules based on the carbazole and sulfone skeletons. This outcome was attributed to the longer excited state lifetimes leading to better interactions with the additives.^[^ ^58^ ^]^

**Figure 14:**
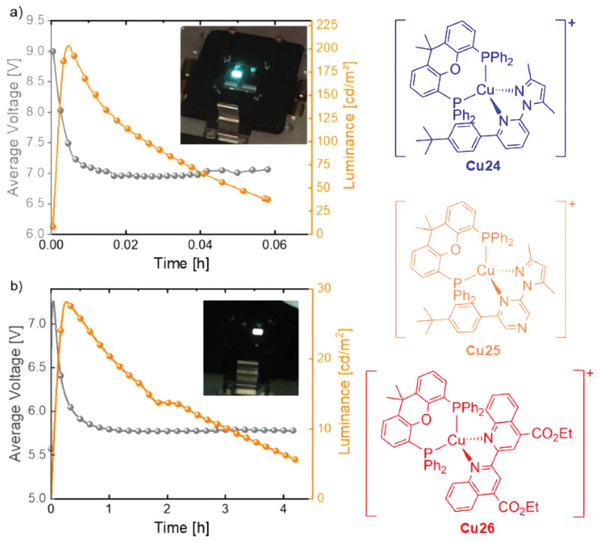
Left: luminance and average voltage of LEC fabricated with 1 and 1:3 in the optimum composition (1:2:3 = 0.977:10^−6^:0.023). Right: Cu(I) complexes used to fabricate blue‐ and white‐emitting LEC. Reproduced with permission.^[^ ^61^ ^]^ Copyright 2022, Wiley.

When 2‐pyridyl pyrazole was employed as an N^N ligand, the energetically higher LUMO determined a shift in the emission maximum to the green‐blue and blue regions in the derived Cu22.^[^ ^59^ ^]^ Similar photoluminescent properties were observed using 3‐(2′‐pyridyl)pyrazole as a chelating ligand. In this case, the corresponding Cu(I) complexes **Cu23** exhibited both TADF and mechanochromism originating from the hydrogen bond between the N‐H moieties and one of the oxygen atoms of the perchlorate counterion. The breaking and restoring of this NH···O bond was reversibly controlled by mechanical grinding and exposure to dichloromethane vapors.^[^ ^60^ ^]^ A similar ligand was employed for the preparation of the first blue‐emitting LEC based on **Cu24** with 3.6 cd A^−1^ of maximum efficiency at 180 cd m^−2^ (see **Figure** **14**a). It is worth mentioning that the fabrication of high‐performing blue‐ and therefore white‐emitting LECs with earth‐abundant metals is challenging. The combination of **Cu24** with **Cu25** and **Cu26** in a tricomponent host:guest system was applied to the first LEC with an overall white emission (CIE coordinates, *x* = 0.30, *y* = 0.35; white light, *x* = *y* = 0.33) and 0.6 cd A^−1^ of maximum efficiency at 30 cd m^−2^ (see Figure 14b). **Cu24** is the host and emits via electrochemiluminescence, while **Cu26** acts as a guest and is excited by hole‐electron recombination or host‐guest energy transfer.^[^ ^61^ ^]^

**Figure 15:**
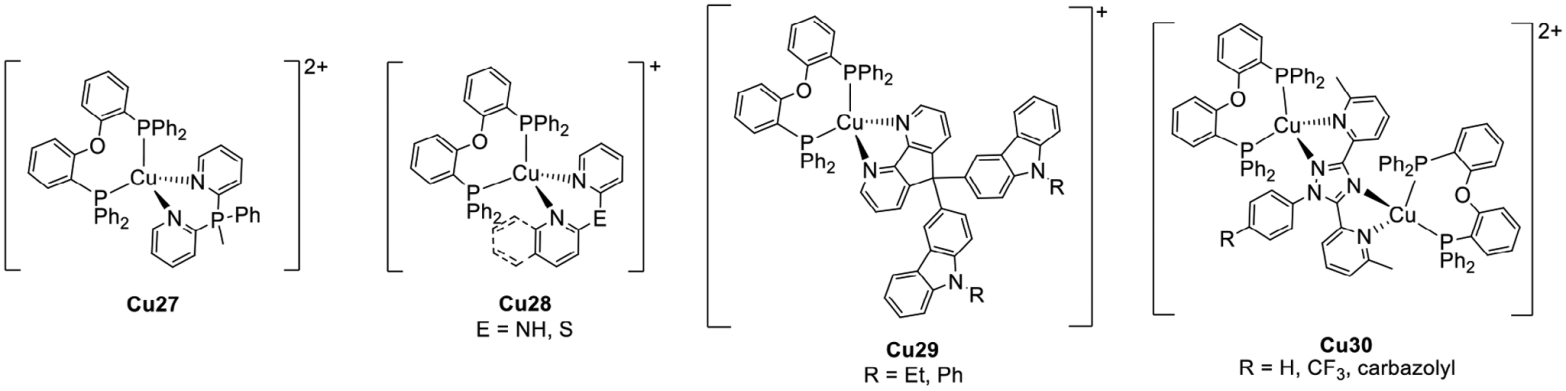
Other cationic Cu(I) complexes exhibiting TADF.

Cu(I) derivatives showing TADF properties were obtained also using pyridyl‐substituted triazoles and di(pyridine‐2‐yl)sulfanes as N^N ligands.^[^ ^62^ ^]^ A phosphonium salt analog to the latter was employed by Costa and coworkers to isolate **Cu27** (see **Figure** **15**), characterized by good electrochemical stability, high ionic conductivity, and TADF properties. The *λ*_max_ was red‐shifted from 583 nm to 606 nm passing from 298 K to 77 K, and the photoluminescent lifetime increased from 6 to 52 µs. The PLQY was 49% as powder and decreased to 29% once **Cu27** was fabricated into a thin film. In the film, the emission maximum was centered at 568 nm and *τ* was ≈5 µs. These properties were exploited to prepare a yellow‐emitting LEC (*λ*_max_ = 536 nm) with improved stability, brightness, and efficiency performances.^[^ ^63^ ^]^ The same group recently described the effect of employing 2‐(pyridine‐2‐yl‐l2‐azanyl)quinoline and 2‐(naphthalen‐2‐ylthio)‐quinoline as N^N ligands (see **Cu28**, Figure 15). The TADF properties and PLQY were enhanced compared to the less π‐extended ligands, but only with 2‐(pyridine‐2‐yl‐l2‐azanyl)quinoline also the corresponding LEC showed improved performances having 0.35 cd A^−1^ efficiency at 117 cd m^−2^ luminance.^[^ ^64^ ^]^

Carbazole‐substituted 4,5‐diazafluorenes were used as N^N ligands to synthesize the yellow‐green emitting Cu(I) complexes **Cu29** (see Figure 15). The luminescence lifetimes and the emission maxima, respectively, passed from 5.7 µs to over 300 µs and from 616 nm to ≈545 nm when cooling down the powder samples from 300 K to 77 K. According to Equation 2, the Δ*E*_ST_ was estimated at ≈725 cm^−1^ (0.09 eV) supporting the TADF mechanism, and the Cu(I) complexes were exploited for the preparation of OLEDs under vacuum‐deposition.^[^ ^65^ ^]^ Compared to other N^N ligands, the carbazole moiety boosted both the photoluminescence and the electroluminescence. The same effect was observed for the binuclear **Cu30** complexes. The binuclear derivatives exhibited green emissions ascribed to TADF at room temperature. The photoluminescent properties were exploited for the preparation of a solution‐processed OLED having EQE of 8.3%, a current efficiency of 27.1 cd A^−1^ and a peak brightness of 2525 cd cm^−2^.^[^ ^66^ ^]^

**Figure 16:**
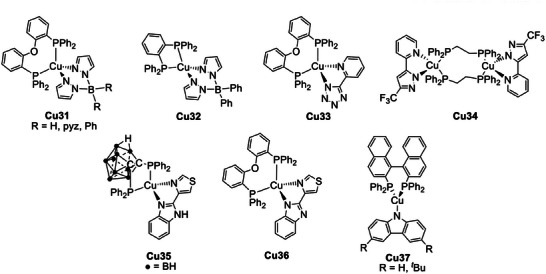
Neutral Cu(I) complexes exhibiting TADF.

Considering neutral Cu(I) complexes, Yersin and coworkers reported the intense blue‐emitting derivatives **Cu31** depicted in **Figure** **16** which exhibited PLQYs up to 90%. The emission was red‐shifted once the complexes were doped in polymethylmethacrylate (PMMA) or dissolved in dichloromethane. This effect was observed together with a decrease in the PLQY and a shortening in the emission lifetime. For instance, [Cu(pz_4_B)(DPEphos)] is characterized by an emission centered at 447 nm with *τ* = 22 µs and 90% PLQY in the solid state and, once dissolved in dichloromethane, *τ* and PLQY were reduced to 0.5 µs and 2%, respectively. When in solution, the molecule is distorted in the excited state leading to vibrational quenching and thus non‐radiative decay paths. Similar considerations can be made for PMMA, however the effect is limited since the matrix is more rigid.^[^ ^67^ ^]^

**Figure 17:**
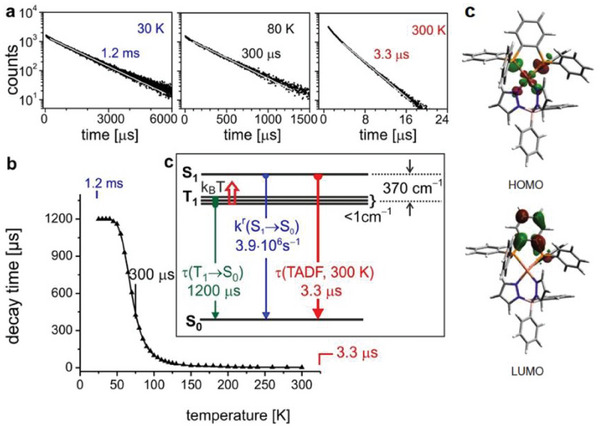
a) Emission decay profile measured for **Cu32** at 30, 80, and 300 K (solid sample, *λ*_ex_ = 378 nm, *λ*_em_ = 540 nm). b) Emission lifetimes *τ* (µs) versus temperature (K). c) HOMO‐LUMO distribution calculated for the optimized triplet state geometry (B3LYP/def2‐SVP). Reproduced with permission.^[^ ^9^ ^]^ Copyright 2017, Wiley.

Complex **Cu32** (see Figure 16) is characterized by one of the smallest Δ*E*_ST_ reported so far, equal to 370 cm^−1^ (0.05 eV). This results in a very short TADF decay of *τ*(TADF) = 3.3 µs, and the emission at 77 K is still dominated by the TADF process, as highlighted in **Figure** **17**a,b. This aspect is also supported by the fact that the emission maxima do not change passing from 300 K to 80 K. Only at temperatures beneath 40 K it was possible to measure the lifetime associated with the emission from the first triplet state *T*_1_, equal to 1.2 ms. In contrast to what was previously described at the beginning of this paragraph, based on TD‐DFT calculations, the HOMO also involves the nitrogen atoms of the pyrazolate moieties, while the LUMO is localized on the *o*‐phenylene ring of the 1,2‐bis(diphenylphosphino)benzene, as highlighted in Figure 17c.^[^ ^31c^ ^]^

**Cu33** (see Figure 16) represents the first complex exhibiting TADF where the ISC was directly observed in the solid state. The derivative is characterized by a bright green emission centered at 512 nm as powder. The 27 ps time constant was measured in the luminescence decay dynamics, and it was attributed to the ISC from *S*_1_ to *T*_1_ (*k*_ISC_ = 3.7 × 10^10^ s^−1^). This value is much lower if compared to Ir(III) derivatives where ISC time constants are ≈100 fs, but can be justified considering the small SOC of Cu compared to Ir (*ξ*_Cu_ = 857 cm^−1^ vs. *ξ*_Ir_ = 3909 cm^−1^).^[^ ^68^ ^]^

The binuclear Cu(I) complexes **Cu34** (see Figure 16) were isolated employing 2‐bis(diphenylphosphino)ethane (dppe) as a P^P ligand and they were characterized by vapochromism as well as TADF properties. The emission changed from yellow to cyan once the dichloromethane‐solvated crystals were exposed to air and the process could be reversed completely using the same solvent. This change in the emission was not detected employing an organic solvent other than dichloromethane.^[^ ^69^ ^]^

*Ortho*‐carborane diphosphines were recently employed by Alconchel and coworkers for the preparation of **Cu35** (see Figure 16). Interestingly, the neutral **Cu35** complex with the *nido*‐carborane diphosphine exhibited TADF properties, while the cationic derivative with the *closo*‐carborane diphosphine was characterized by thermochromism.^[^ ^70^ ^]^ It is worth mentioning that Cu(I) complexes bearing *nido*‐carborane diphosphines and exhibiting TADF properties were previously reported in combination with substituted bpy and phen ligands, and applied in solution‐processed OLEDs.^[^ ^71^ ^]^ The photoluminescent properties of **Cu35** were investigated in the solid state at different temperatures revealing *τ*(*S*_1_) = 0.26 µs, *τ*(*T*_1_) = 2.92 ms, and Δ*E*_ST_ = 925 cm^−1^ (0.11 eV) according to Equation 2. Similar values were obtained when **Cu35** (5 wt.%) was doped in a PMMA matrix. The mechanism responsible for the emission was ascribed to an MLL'CT transition for both the complexes but the different photoluminescent behavior was attributed to the lower electron‐withdrawing effect on the *nido*‐carborane diphosphine compared to the *closo*‐carborane analog. Based on DFT calculations, the orbitals implicated in the emission of **Cu35** are characterized by a lower contribution of the metal center and the diphenyl rings of the P^P ligand, and a higher involvement of the carborane cage.^[^ ^70^ ^]^ The same authors recently reported the complex **Cu36** (see Figure 16), which exhibited TADF properties despite the low quantum yield. The analogous Cu(I) complex having Xantphos as the chelating diphosphine was not emissive at room temperature, but blue luminescence was observed at 77 K. For **Cu36** *τ*(DF) and *τ*(*T*_1_) were respectively equal to 757 µs and 19.1 ms, thus the Δ*E*_ST_ was estimated ≈455 cm^−1^ (0.06 eV). The different emissive properties compared to **Cu35** can be rationalized considering that the negative charge is localized on the N^N ligand and not on the P^P, thus originating different frontier orbitals.^[^ ^72^ ^]^

To conclude, Steffen and coworkers recently reported the three‐coordinated neutral Cu(I) complexes **Cu37** (see Figure 16) having an enantiomerically pure 2,2′‐bis(diphenylphosphino)−1,1′‐binaphthyl (R/S‐BINAP) as acceptor and a carbazolate as donor. The emission was ascribed to a ^1/3^LLCT and the derivatives were characterized by circularly polarized luminescence (CPL) with dissymmetry factors *g*_lum_ up to ±2.1 × 10^−2^ in the solid state, as well as TADF properties. In addition, **Cu37** as single crystals exhibited mechanochromic properties due to the disruption of intermolecular C‐H···π interactions, with a red shift in the emission between 25 and 57 nm.^[^ ^73^ ^]^

#### 4.3.3 C‐donor Ligands

**Figure 18:**
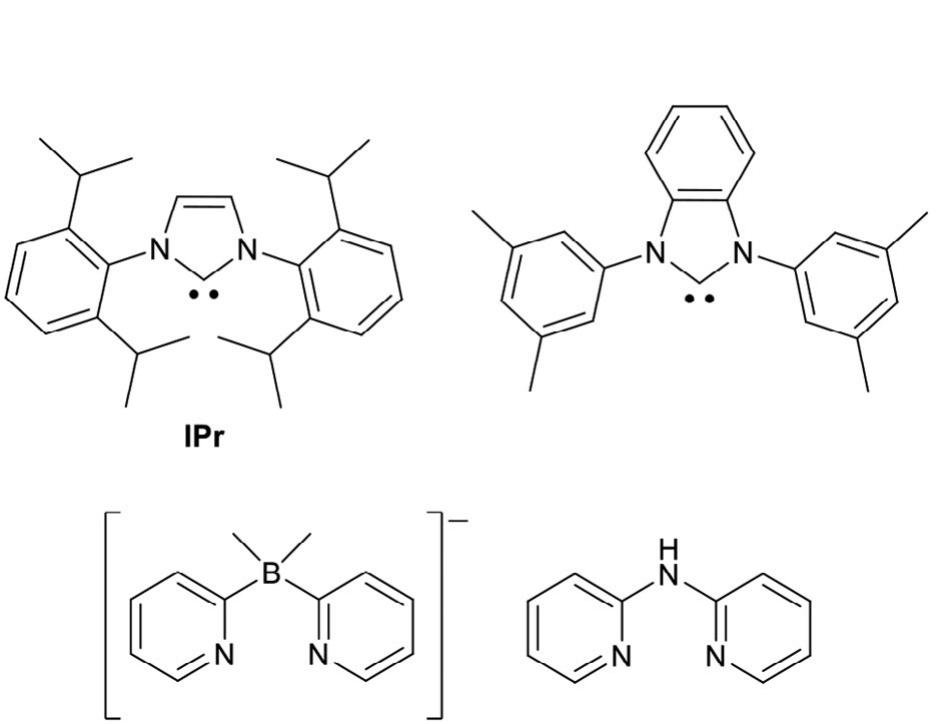
First carbenes and chelating N^N ligands were tested for the preparation of Cu(I) complexes exhibiting TADF.

The most common C‐donor ligands applied for the synthesis of TADF Cu(I) complexes are N‐heterocyclic carbenes (NHCs), which behave as strong *σ*‐donors and weak π‐acceptors.^[^ ^74^ ^]^ Three‐coordinated Cu(I) complexes were isolated in combination with dimethyldi(pyridine‐2‐yl)borate acting as N^N ligand. Despite the Cu(I) species with both the carbenes depicted in **Figure** **18** exhibiting bathochromism and elongation of the photoluminescent lifetime at 77 K, only the Cu(I) complex containing IPr showed TADF properties. This aspect was rationalized considering that the Δ*E*_ST_ increases from 740 (0.09 eV) to 3000 cm^−1^ (0.37 eV) changing the position of the alkyl substituents from the *ortho*‐ to the *meta*‐positions due to the different torsion angles of the fragment N_NHC_‐C_NHC_‐Cu‐N_py_.^[^ ^10^ *^,^* ^75^ ^]^ Therefore, the investigation on Cu(I) complexes exhibiting TADF is mostly limited to the 2,6‐isopropenylphenyl substituted carbene **IPr**.

**Figure 19:**
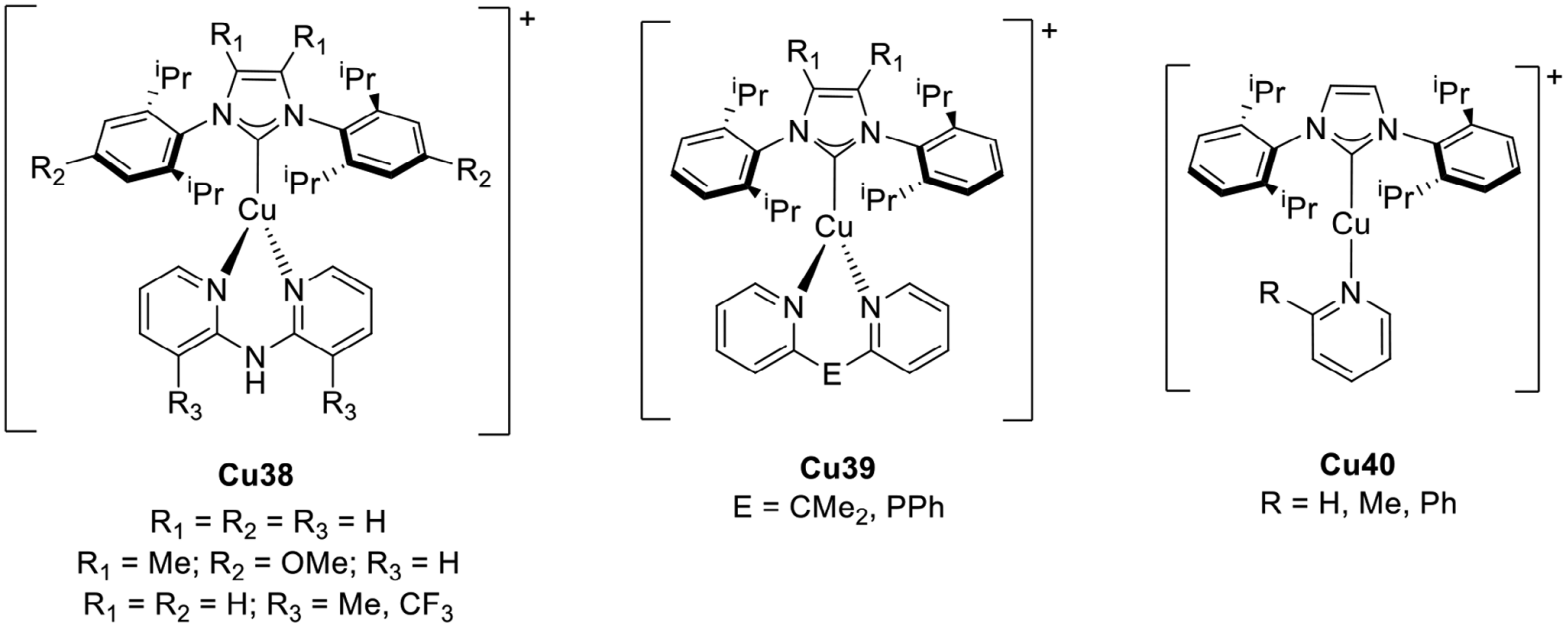
Cationic carbene Cu(I) complexes exhibiting TADF.

Instead of the borate anion, Costa and coworkers employed the neutral di(pyridin‐2‐yl)amine (py_2_NH) as the N^N ligand to isolate the Cu(I) complexes **Cu38** depicted in **Figure** **19**. The introduction of electron‐donating (e.g., Me orOMe) or withdrawing (e.g., CF_3_) groups on the py_2_NH skeleton provoked the hypsochromic or bathochromic shift of the emission maxima, respectively. Complex **Cu38** having **IPr** and py_2_NH as ligands was applied for the development of the first blue‐emitting Cu(I)‐derived LECs that achieved luminance and current efficiency comparable to the devices prepared with Ir(III) complexes. However, the performances dropped under repetitive current‐voltage‐luminance sweeps between 0 and 11 V due to the degradation of the emitter.^[^ ^76^ ^]^ One of the main problems of these devices was the degradation of the emitter using the solvents commonly employed for the LEC fabrication with the formation of oxidized species that can act as carrier trappers and luminance quenchers. However, it was observed that adding ionic additives and 4,4′‐bis(*N*‐carbazolyl)−1,1′‐biphenyl (CBP) as hole transporter could limit this issue. Thus, the performances of the LEC containing the previously described **Cu38** having IPr and 2,2′‐bis‐(3‐methylpyridyl) amine as ligands were optimized. In particular, the maximum luminance increased from 20 to 160 cd m^−2^, and the efficiency improved from 0.17 to 1.2 cd A^−1^.^[^ ^77^ ^]^ When 2,2‐(2,2′‐dipyridyl)propane (py_2_CMe_2_) and phenyl‐2,2′‐dipyridylphosphine (py_2_PPh) were used as N^N (see **Cu39**, Figure 19), the structure of the bipyridyl ligand changed from planar to boat‐like, causing an enhancement of the PLQY without altering the TADF properties, as well as the increase of the ionic conductivity of thin films. Both aspects significantly improved the performances of the derived LECs (70 nm PEDOT:PSS, 90 nm electroluminophore, 90 nm Al) compared to previously reported devices in terms of luminance (10‐15 cd m^−2^) and efficiency (0.2–0.4 cd A^−1^) under 0.5 mA of current.^[^ ^78^ ^]^

Steffen and coworkers described the first example of linear TADF Cu(I) complexes (see **Cu40**, Figure 19) whose emission was ascribed to ^3^MLCT. The derivatives were only scarcely emissive as single crystals; however, they showed an intense blue to blue‐green luminescence with PLQYs up to 87% and microsecond‐long lifetimes as powders, in 2‐methyl THF at 77 K, and once fabricated into neat or doped PMMA films (1–10 wt.%). The emission was ascribed to the formation of cation–anion Cu···F interactions that are negligible in single crystals but determine a high *k*_r_ in powders and PMMA matrices. The same effect could be achieved by grinding the single Cu(I) crystals, leading to a stimulus‐responsive material.^[^ ^79^ ^]^

Concerning neutral species, luminescent linear carbene‐copper‐amide complexes are an emerging class of materials to be applied in optoelectronics, in particular OLEDs. In this type of derivative, the emission is typically an LLCT since it usually takes place from the π‐donating amide to the π‐accepting carbene through the *d* orbitals of the metal center, similar to what is commonly observed in organic TADF molecules. Thus, the HOMO and the LUMO are respectively distributed on the amide and the carbene ligands, with scarce involvement of the Cu(I) center. The coplanar geometry of this class of complexes maximizes the long‐range coupling between the *p* orbitals of the amide and the carbene, determining high *k*_r_, and thus large oscillator strengths in combination with sub‐µs DF lifetimes.^[^ ^80^ ^]^

**Figure 20:**
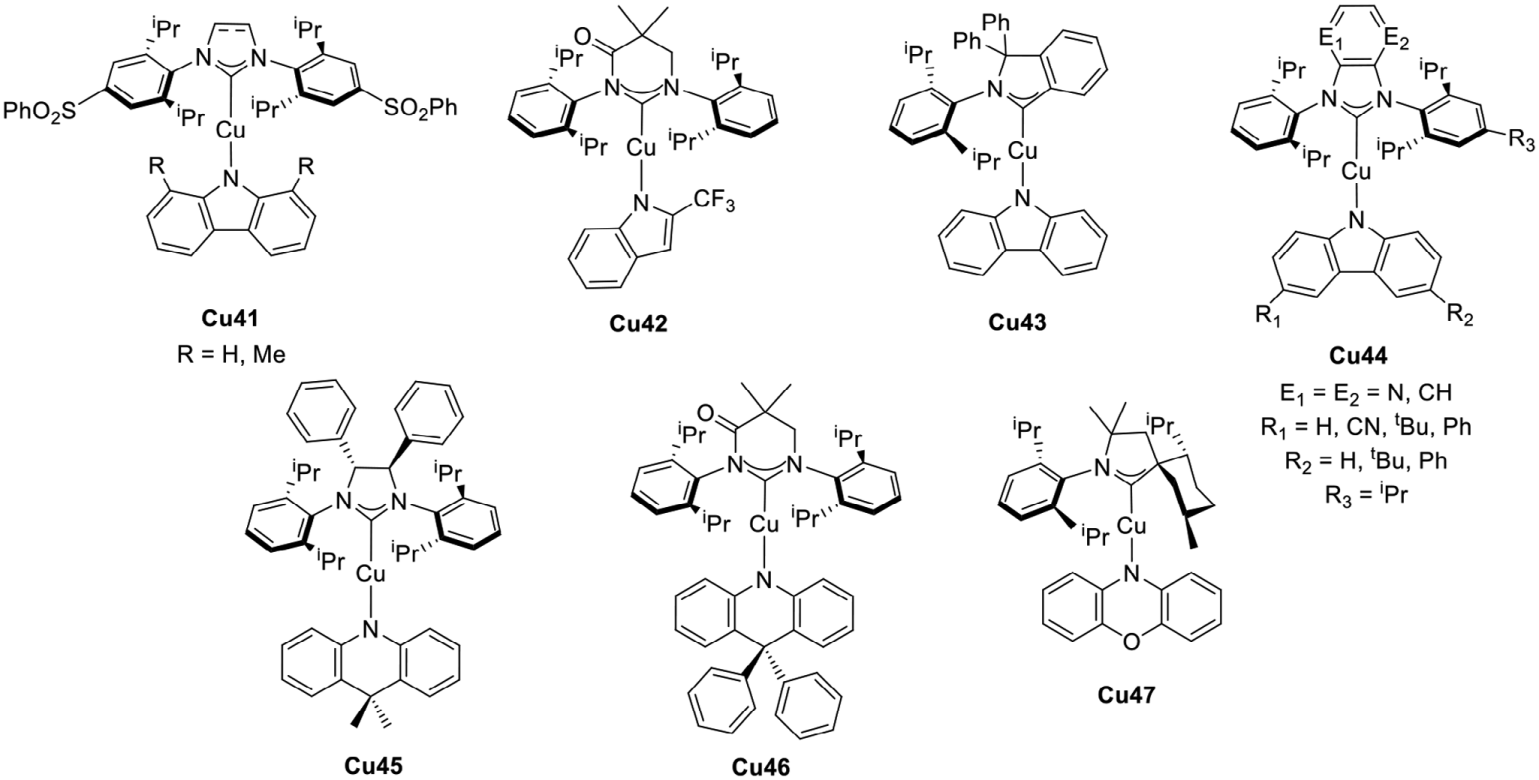
Selected examples of carbene‐Cu(I)‐amide complexes exhibiting TADF.

Even though [Cu(IPr)(Cbz)] (Cbz = carbazolate) exhibited only ultra‐long phosphorescence at room temperature,^[^ ^81^ ^]^ similar derivatives showed TADF properties. Replacing the NHC with a cyclic (alkyl)(amino)carbene (cAAC) stabilized the empty *p* orbitals of the carbenic carbon, thus enabling the observation of DF and the charge transfer nature of the emission.^[^ ^80^ ^]^ The introduction of a phenylsulfonyl acceptor in the cAAC skeleton resulted in a PLQY up to 90% and *k*_r_ ≈ 3 × 10^5^ s^−1^ in the derived **Cu41** (see **Figure** **20**). This design strategy combines a low Δ*E*_ST_ with a small SOC due to the presence of the metal center.^[^ ^82^ ^]^

Recently it was observed by Li et al. that a confined configuration of the amide between coplanar (large Δ*E*_ST_, high *k*_r_) and orthogonal (small Δ*E*_ST_, low *k*_r_) with respect to the metal center and the carbene could balance the Δ*E*_ST_ and the *k*_r_. When a twisted configuration with a dihedral angle of ≈45° was obtained the corresponding blue‐emitting **Cu42** exhibited PLQY up to 74% and short TADF lifetimes (≈1.9 µs). These promising photoluminescent properties were exploited for the preparation of vacuum‐deposited OLEDs with EQE over 20% and 10% in doped and hosted‐free emitting layers, respectively.^[^ ^83^ ^]^

Another effective strategy to stabilize the empty *p* orbitals consists of introducing extended π‐conjugated systems or electron‐withdrawing groups such as carbonyl moieties in the carbene skeleton.^[^ ^80^ ^]^ Employing a cyclic (amino)(aryl)carbene (CAArC), the corresponding **Cu43** showed a deep‐red TADF emission with *λ*_max_ = 621 nm and PLQY = 32%. The radiative rate constant *k*_r_ was estimated ≈9 × 10^5^ s^−1^, which is higher than the values commonly observed in Ir(III) and Pt(II) species.^[^ ^84^ ^]^ Similarly, introducing a bulky pyrazine‐ or pyrazine‐fused NHC in combination with Cbz determined enhanced bonding interactions in the corresponding **Cu44**. Blue to red TADF emissions with *k*_r_ between 1.1 and 2.2 × 10^6^ s^−1^, and PLQYs up to 89% were detected by Che et al. and ascribed to LLCT transitions. **Cu44** was employed for the preparation of vapor‐deposited OLEDs with EQE and luminance up to 23.6% and 222 200 cd m^−2^, respectively. The devices exhibited also an extremely long device lifetime, reaching 1300 h at 1000 cd m^−2^.^[^ ^85^ ^]^

When a chiral carbene was employed as an acceptor, the two derived **Cu45** enantiomers exhibited aggregation‐induced CPL due to a large dissymmetry factor (*g*_lum_ = 0.027). This feature originates from the limited ligand‐ligand rotation and the helical packing of the enantiomers in the crystals. In addition, Cu45 was characterized by aggregation‐induced TADF properties with Δ*E*_ST_≈ 750 cm^−1^ (0.09 eV) based on the lifetimes at 77 k ( *τ* = 316 µs) and 300 K ( *τ* = 0.62 µs). The photophysical properties changed depending on the aggregation state in terms of emission maxima and delayed fluorescence lifetimes.^[^ ^86^ ^]^ The similar **Cu46** reported by Ying and colleagues exhibited an intense red emission (*λ*_max_ ≈ 630 nm) and TADF properties with a PLQY over 70%, which resulted in an EQE of 21.1% in the corresponding OLED. The device exhibits one of the best performances reported so far employing coinage metal complexes.^[^ ^87^ ^]^

**Figure 21:**
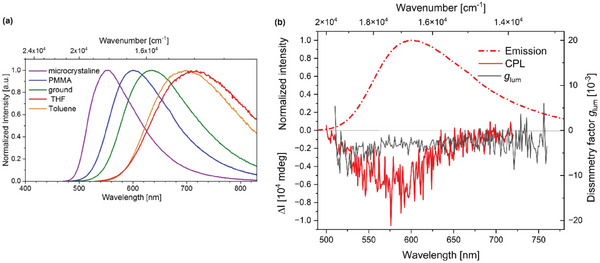
a) Normalized PL emission spectra of **Cu47** in various media under argon atmosphere and at room temperature. b) Normalized PL spectrum (dotted red), CPL spectrum (solid red), and corresponding g_lum_ values (grey) of **Cu47** in PMMA at room temperature. Reproduced with permission.^[^ ^88^ ^]^ Copyright 2023, Wiley.

Mechanochromic properties were also recently observed by Steffen and coworkers for a series of linear Cu(I) complexes with chiral cAAC. Complex **Cu47** (depicted in **Figure** **21**) exhibited CPL being characterized by luminescence dissymmetry factor *g*_lum_ = 3.4 × 10^−3^ in PMMA films and an orange‐red TADF emission with *k*_r_ = 6.7 × 10^5^ s^−1^ originating from ^1/3^LLCT states. In contrast to the other Cu(I) complexes described, CPL was detected in **Cu47** due to the combination of butterfly distortion of the N‐donor ligand and the hindered rotation of the cAAC in a rigid matrix.^[^ ^88^ ^]^

#### 4.3.4 Other Ligands

**Figure 22:**
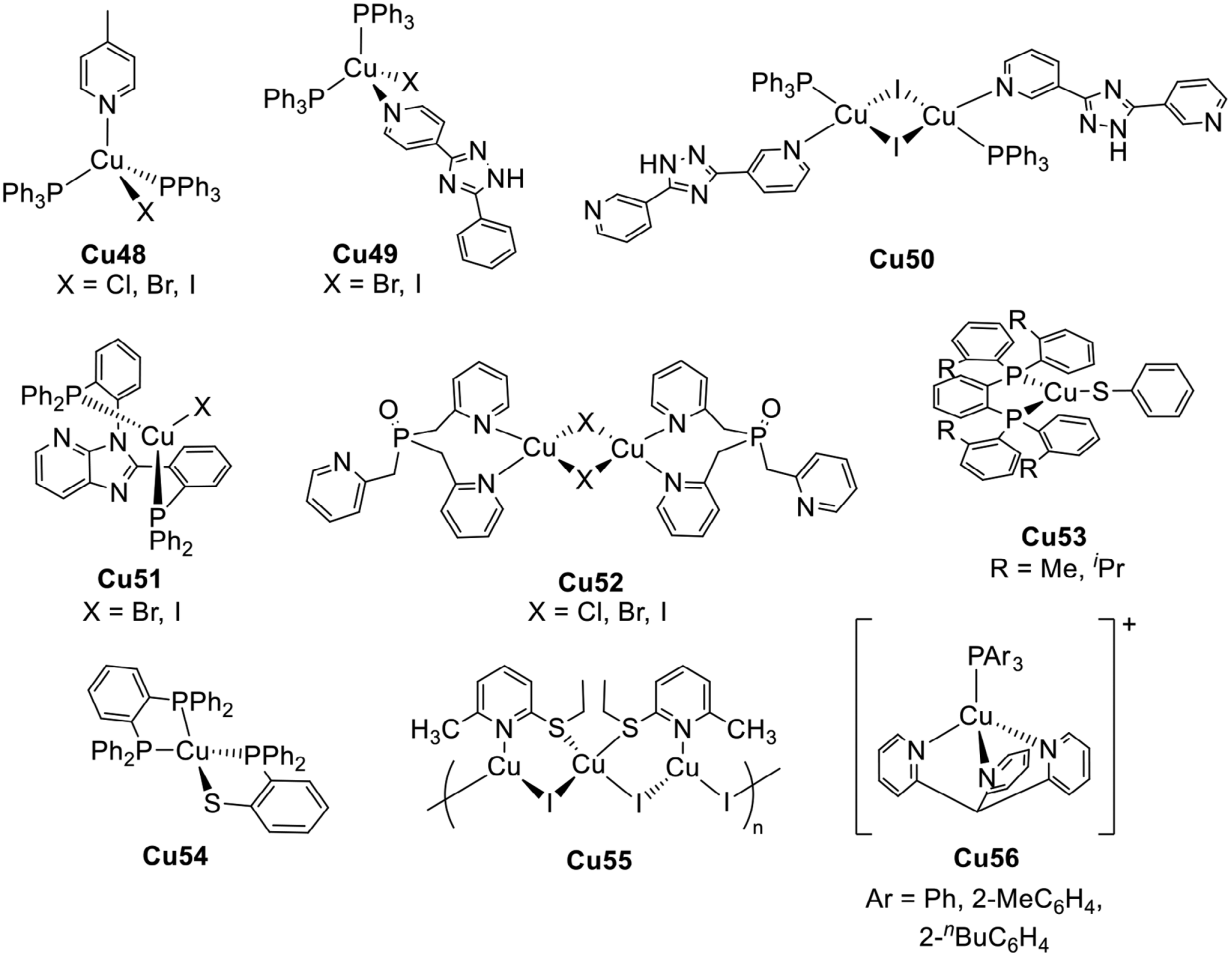
Luminescent Cu(I) complexes exhibiting TADF.

**Figure 23:**
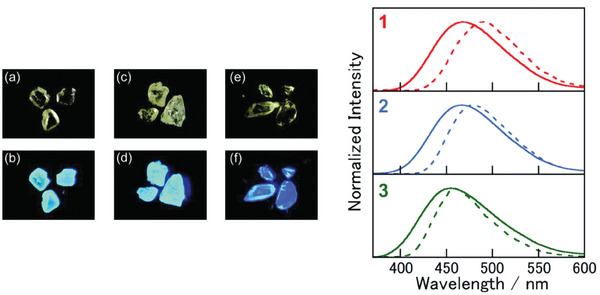
Left: pictures of crystalline **Cu48**, X = Cl (a and b), Br (c and d), I (e and f) under bright field and UV light. Right: normalized PL spectra at the solid state (*λ*_ex_ = 350 nm) at room temperature (solid line) and 77 K (dotted line). Reproduced with permission.^[^ ^89^ ^]^ Copyright 2014, Royal Society of Chemistry.

Although the majority of luminescent Cu(I) complexes exhibiting TADF properties belong to the previously described groups, a small number of examples with different ligands have been reported. For instance, 4‐methylpyridine was used for the preparation of **Cu48** (see **Figure** **22**). As observable in **Figure** **23**, the complexes were brightly blue‐emissive with PLQY almost reaching 100% for the chloro‐ and bromo‐species at room temperature. Based on Equation 2, the Δ*E*_ST_ was estimated between 940 cm^−1^ (0.12 eV) and 1170 cm^−1^ (0.15 eV) for **Cu48**, together with *τ*(*S*_1_) and *τ*(*T*_1_) respectively in the range 14–47 ns and 34–50 µs.^[^ ^89^ ^]^

Similarly, 3‐phenyl‐5‐(pyridin‐4‐yl)−1,2,4‐triazole was employed for the synthesis of **Cu49** (see Figure 22) in combination with triphenylphosphine and halides, i.e., Br and I. The derivatives were characterized by intense blue photoluminescence in solution and green emissions in the solid state with PLQYs up to 100% at 77 K. Mechanochromism was also observed upon grinding, with the emission changing from green to orange. The process could be reversed by heating the samples or exposing them to acetonitrile vapors. **Cu49** was applied for the preparation of a green‐emitting OLED with an EQE of up to 13.5% in the case of the bromo species.^[^ ^62a^ ^]^ Similar photoluminescent properties were described by the same authors for the binuclear **Cu50** having another pyridyltriazole derivative (see Figure 22). The energy gap Δ*E*_ST_ between the first triplet and singlet states (*T*_1_ and *S*_1_) was estimated at ≈962 cm^−1^ (0.12 eV) based on Equation 2, with lifetimes of 134 and 10.7 µs, respectively.^[^ ^90^ ^]^

**Figure 24:**
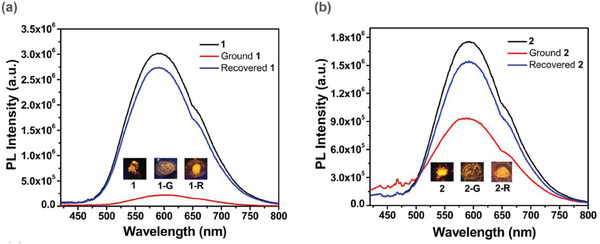
Emission spectra from **Cu51** exhibiting crystallization‐enhanced TADF properties. Reproduced with permission.^[^ ^91^ ^]^ Copyright 2022, Elsevier.

The orange‐emitting three‐coordinated Cu(I) derivatives **Cu51** exhibiting TADF features were isolated by reacting a rigid chelating phosphine based on the pyridoimidazole fragment with Cu(I) halides. In this case, the photophysical properties could be enhanced by crystallization due to the formation of CH···π interactions in the molecular packing structures. As highlighted in **Figure** **24**, the intensity of the PL spectra significantly varies passing from the crystalline to the ground samples. The recrystallization of **Cu51** by exposure to acetonitrile restored the luminescence intensity.^[^ ^91^ ^]^

Differing from the N^P ligands presented above, phosphine oxides such as tris(pyridin‐2‐ylmethyl)phosphine oxide acted as bidentate ligands in the corresponding binuclear **Cu52** complexes (see Figure 22). The blue‐green TADF emission was attributed to a (M+X)LCT emission with PLQY up to 54%. The X‐ray radioluminescence of **Cu52** was investigated, revealing the opposite trend compared to the photoluminescent features. The emissive properties and the bathochromic shift increase passing from I to Br and Cl, while X‐ray irradiation is absorbed more efficiently by heavier atoms.^[^ ^92^ ^]^

Osawa and coworkers reported the use of thiolates as anionic ligands instead of halides. In the solid state, the corresponding trigonal **Cu53** complexes (see Figure 22) were characterized by blue‐green TADF properties with PLQYs of 95% both at 293 and 77 K. The photoluminescent lifetime *τ* was between 5.0 and 6.6 µs at 293 K, while at low temperature it was over 1 ms long. In both cases, the Δ*E*_ST_ was estimated under 700 cm^−1^ (0.09 eV). In 2‐methyl THF solution, the emission was bathochromically shifted to the orange region of the spectrum, with a consequent decrease in both PLQY and lifetimes.^[^ ^93^ ^]^ The same author employed the bidentate 2‐diphenylphosphinobenzenethiolate as a P^S ligand (see **Cu54**, Figure 22). Its strong electron‐donating character lowers the MLCT character in the excited state, reducing the contribution of the metal orbitals to the HOMO. The green TADF emission was centered at 521 nm and ascribed to an LLCT mechanism based on DFT calculations. The intriguing photophysical properties were exploited for the preparation of a green‐emitting OLED characterized by a current efficiency of 21.3 cd A^−1^ and an EQE of 7.8%.^[^ ^94^ ^]^

**Figure 25:**
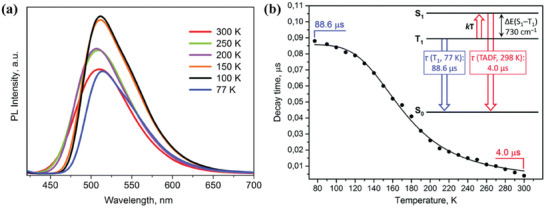
a) Temperature‐dependent PL spectra of complex **Cu55** (*λ*_ex_ = 380 nm); b) Emission lifetime *τ* versus temperature (*λ*_ex_ = 380 nm; *λ*_em_ = 515 nm). Reproduced with permission.^[^ ^95^ ^]^ Copyright 2019, Royal Society of Chemistry.

A mixed N^S ligand was employed by Artem'ev and coworkers for the preparation of a monodimensional coordination polymer **Cu55** having the general formula [Cu_3_I_3_L_2_]_n_ where L is 2‐(ethylsulfanyl)−6‐methylpyridine (see Figure 22). This product could only be obtained by a mechanochemical route in the presence of a few drops of acetonitrile. Under neat conditions or in solution, the complexes [Cu_2_I_2_L_2_] and [Cu_3_I_3_L_2_]_n_ were isolated, respectively. As highlighted in **Figure** **25**, **Cu55** exhibits blue‐green TADF properties ascribed to a (M+X)LCT mechanism. Passing from 298 to 77 K the emission lifetime *τ* increased from 4 to 88.6 µs, and the Δ was estimated ≈730 cm^−1^ (0.09 eV) based on Equation 2.^[^ ^95^ ^]^

Finally, TADF properties were also detected when a tripodal N^N^N ligand such as tris(2‐pyridyl)methane was employed in combination with an aromatic phosphine for the synthesis of the deep blue‐emitting **Cu56** complexes (see Figure 22). The counterion plays an important role in the photoluminescence of this Cu(I) species, particularly in the PLQY (43% for PF_6_^−^ vs. 7% for BPh_4_^−^), by influencing the molecular packing in the solid state. The PLQY dramatically decreases changing triphenylphosphine with *o*‐substituted P^P ligands. Although TADF is observed, phosphorescence is still predominant in the emission at room temperature (60% vs. 40%).^[^ ^96^ ^]^

### 4.4 Group 11: Ag(I) Complexes

Besides Cu, which is the most prevalent coinage metal, Ag and Au have extensively been applied as luminescent complexes for a wide variety of applications. Nevertheless, Ag(I) and Au(I) complexes exhibiting TADF properties are much less well explored than their Cu(I) counterparts. Although the metal center is not truly earth‐abundant (its natural abundance is 0.08 ppm), in this paragraph we will present Ag(I) complexes to offer a direct comparison with the design strategies used in Cu(I) complexes. As the natural abundance of Au is even lower on the earth's crust (0.004 ppm) the corresponding complexes will not be discussed.

The major challenge for Ag(I) complexes is presented by the higher ionization potential of the metal, often exhibiting PF and/or phosphorescence from a metal‐perturbed intra‐ligand excited state due to the heavy‐atom effect. The full shell of the 4*d* orbitals usually has a lower energy than the occupied orbitals of the ligands, where the HOMO of the corresponding complex is localized. Since it was observed that the *S*_1_ and *T*_1_ states should present a robust charge transfer character, the strategic generation of low‐energy MLCT excited state in Ag(I) complexes was achieved by destabilization of the low‐lying 4*d* orbitals. Through the involvement of electron‐donating phosphines in the coordination sphere, the aim of a small Δ*E*_ST_ separation and thus TADF could be achieved.^[^ ^6^ ^]^

**Figure 26:**
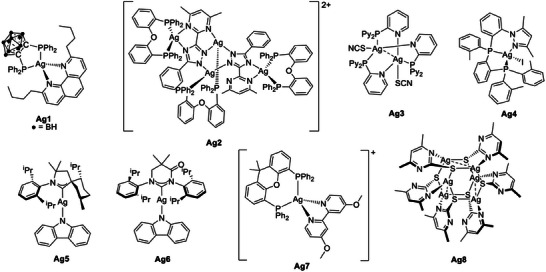
Luminescent Ag(I) complexes exhibiting TADF.

**Figure 27:**
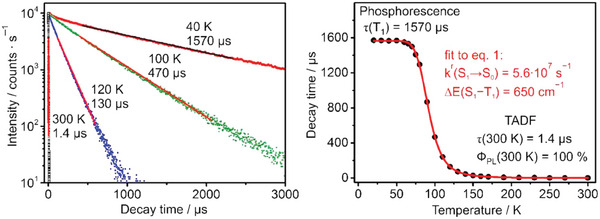
Luminescence decay lifetimes of **Ag1** (powder sample) measured at different temperatures (left) and plotted versus temperature (right). Reproduced with permission.^[^ ^97^ ^]^ Copyright 2017, Royal Society of Chemistry.

This design was followed by Yersin, Artem'ev, and coworkers. One of the first examples with outstanding properties was the tetracoordinated Ag(I) complex Ag1 (see **Figure** **26**) having 2,9‐di‐*n*‐butyl‐1,10‐phenanthroline and the chelating *nido*‐carborane bis(diphenyl)phosphine as ligands. This chelating phosphine is a strong electron donor, being also negatively charged. The destabilization of the metal‐centered orbitals generated a metal‐ligand‐to‐ligand charge transfer (MLL'CT) as the lowest excited state, from which the RISC process established the repopulation of the singlet state *S*_1_ from the triplet *T*_1_. In particular, the Δ*E*_ST_ was experimentally determined ≈650 cm^−1^ (0.08 eV). Photophysical investigations showed that the compound as powder emits at 526 nm with an exceptional PLQY of 100%. The decay time is monoexponential and subject to a tremendous increase at low temperatures, going from 1.4 µs at 300 K to 1300 µs at 77 K. At lower temperatures, from 60 K to 20 K, phosphorescence is responsible for the decay, and the lifetime remains constant at 1570 µs (**Figure** **27**).^[^ ^97^ ^]^

A similar design was employed to prepare the tetranuclear Ag(I) complex **Ag2** with 4,6‐dimethyl‐2‐(5‐phenyl‐4*H*‐1,2,4‐triazol‐3‐yl)pyrimidine and DPEphos as bridging ligands. The emission in the solid state is centered at 527 nm with a PLQY of 76% at room temperature, while it is slightly red‐shifted at 77 K (*λ*_max_ = 529 nm) and the PLQY increases up to 90%. **Ag2** is characterized by *τ*(TADF) = 0.65 µs at room temperature, which corresponds to a *k*_r_ of 1.2 × 10^6^ s^−1^, one of the highest values reported so far for TADF materials.^[^ ^98^ ^]^

The complex **Ag3** was prepared using the previously described tris(2‐pyridyl)phosphine (py_3_P), which chelates the silver core with the P‐atom and the N‐atom of one of the three pyridine moieties. By employing non‐coordinating solvents such as chloroform or dichloromethane in the synthesis, the isolated complex was dinuclear, having a thiocyanate per silver atom to equilibrate the charges. This dinuclear complex exhibited a strong sky‐blue photoluminescence with an emission maximum centered at 483 nm when solvated with dichloromethane, and at 478 nm when solvated with chloroform. The PLQY is ≈70% at 300 K, while the non‐solvate Ag(I) complex only showed a 16% quantum yield. The presence of solvent molecules in the crystal lattice of **Ag3** allowed an increased rigidity of the system and a reduced flattening distortion. Upon cooling to 77 K, a bathochromic shift of the emission maximum occurs, along with an increase of the PLQY, which reaches 100%, while the emission decay increases from 12 µs to ≈100 µs. Temperature dependence of the excited state lifetime graphs below 77 K was not necessary, as already from 300 K to 77 K the fitting curve showed that **Ag3** exhibits phosphorescence in the temperature range from 125 K to 77 K, while when increasing the temperature, a thermal population of the *S*_1_ occurs. Further support of a room temperature TADF emission was given by DFT and TD‐DFT, where Δ*E*_ST_ was estimated at ≈970 cm^−1^ (0.12 eV).^[^ ^99^ ^]^ In contrast to Cu(I) complexes, when PyrPhos and arsine species such as py_2_PhAs and py_3_As were employed as ligands, the corresponding Ag(I) mono‐ and polynuclear derivatives did not exhibit photoluminescent properties.^[^ ^43^ *^,^* ^100^ ^]^

The tridentate N,P,P‐ligand based on diphosphanyl‐pyrazole employed by Klein and coworkers for the preparation of Cu(I) complexes was used also to isolate the corresponding Ag(I) iodo‐complex **Ag4**. The powder emission at 300 K has an MLCT nature and is blue‐shifted compared to the Cu(I) analog (*λ*_max_ = 530 nm), being centered at 479 nm with a photoluminescent lifetime of 13 µs and a PLQY of 70%. At 77 K the emission maximum is shifted bathochromically to 484 nm and the lifetime has a biexponential decay with *τ*_1_ = 270 µs and *τ*_2_ = 470 µs, supporting the TADF mechanism.^[^ ^45^ ^]^

Linear Ag(I) coordination compounds with two monodentate ligands such as carbenes and amides also showed promising results. Similar to the Cu(I) analogs, the HOMO is localized on the amide‐ligand, while the LUMO is carbene‐centered. This strategy was followed independently by Thompson, Nolan, Steffen, and coworkers. The complexes **Ag5** and **Ag6** reported by Hamze et al. exhibit an intense photoluminescence both in 2‐methyl THF and in a polystyrene film with 1 wt.% of emitter. In particular, **Ag5** emits at 512 nm and has a PLQY of 70% in solution, which increases up to 100% in the polymer matrix, while the emission profile of **Ag6** is bathochromically shifted to 568 nm and the PLQY drops down to 6% in solution, but it reaches ≈80% in the polystyrene film. It is worth mentioning that the measured *τ* was in the range of 300–500 ns for these Ag(I) compounds while the corresponding Cu(I) and Au(I) complexes, had lifetimes in the microsecond range. Investigations at low temperatures revealed that above 200 K the emission has a 95% TADF character and the measured Δ*E*_ST_ are 1613 cm^−1^ (0.20 eV) for **Ag5** and 887 cm^−1^ (0.11 eV) for **Ag6**.^[^ ^101^ ^]^

All the Ag(I) complexes presented so far are neutral compounds, suitable for OLED applications, but not for LECs, where the ionic nature of the compound is a main requirement. Recently, an efficient LEC device was prepared by Viciano‐Chumillas, Costa, and coworkers. The design of the Ag(I) complex **Ag7** resembles the strategy used in the Cu(I) heteroleptic derivatives previously described. The Ag(I) metal center is coordinated by two neutral chelating ligands, i.e., 4,4′‐dimethoxy‐2,2′‐bipyridine and Xantphos, leading to a cationic complex. In thin films, the Ag(I) derivatives have a PLQY of 20%, with the emission maximum centered at 520 nm and ascribable to a ^3^MLCT. Unfortunately, the authors did not investigate further if the compound exhibited TADF properties. Despite the optimization of the LEC device, the maximum luminance and the efficiency were up to 40 cd m^−2^ and 0.2 cd A^−1^, respectively, with stability of only 30 seconds when BF_4_^−^ was used as counterion. The degradation mechanism was attributed to the formation of Ag nanoclusters upon electron injection, leading to a quick deterioration of the emitting region. When PF_6_^−^ was the counterion, a better‐performing device could be fabricated with a double‐layered architecture, reaching a maximum luminance of 131 cd m^−2^, efficiency of 0.6 cd A^−1^, and stability for >80 h.^[^ ^102^ ^]^

To conclude, the green emitting hexanuclear Ag(I) cluster **Ag8** characterized by TADF properties and ≈92% PLQY was recently employed for high‐resolution X‐ray imaging, achieving sensitivity ≈22 times higher than the ones described for common TADF organic scintillator materials. In contrast to the other examples presented here, the emission maximum is blue‐shifted upon cooling the sample to 77 K, suggesting an increase in energy as commonly observed in ^3^CC (cluster‐centered) state phosphorescence emissions. With *τ*(*S*_1_) = 152 ns and *τ*(*T*_1_) = 22 µs, the Δ*E*_ST_ was estimated at ≈1050 cm^−1^ (0.13 eV) based on Equation 2.^[^ ^103^ ^]^

### 4.5 Group 12: Zn(II) Complexes

As Zn(II) is a post‐transitional element, *d*‐*d* transitions are not expected due to the filled 3*d* shell. Therefore, Zn(II) complexes are normally characterized by a narrow and absorption‐mirrored emission from the lowest singlet excited state *S*_1_, which is thus ascribed to fluorescence. The role of the metal center is to provide stability to the ligand at the excited state.

Nevertheless, the growing interest in earth‐abundant metal‐based emitters for optoelectronic applications led to finding design principles for TADF emitters based on Zn(II) complexes.^[^ ^104^ ^]^ In particular, the ability of Zn(II) complexes to exhibit TADF is attributed to the specific ligand coordination and the involvement of different excited electronic states, as will be further discussed below. The spin‐orbit coupling mediated by the zinc(II) ion is weak; however, it is sufficient to enable the intersystem crossing mechanism, populating the singlet excited state by RISC.^[^ ^105^ ^]^

**Figure 28:**
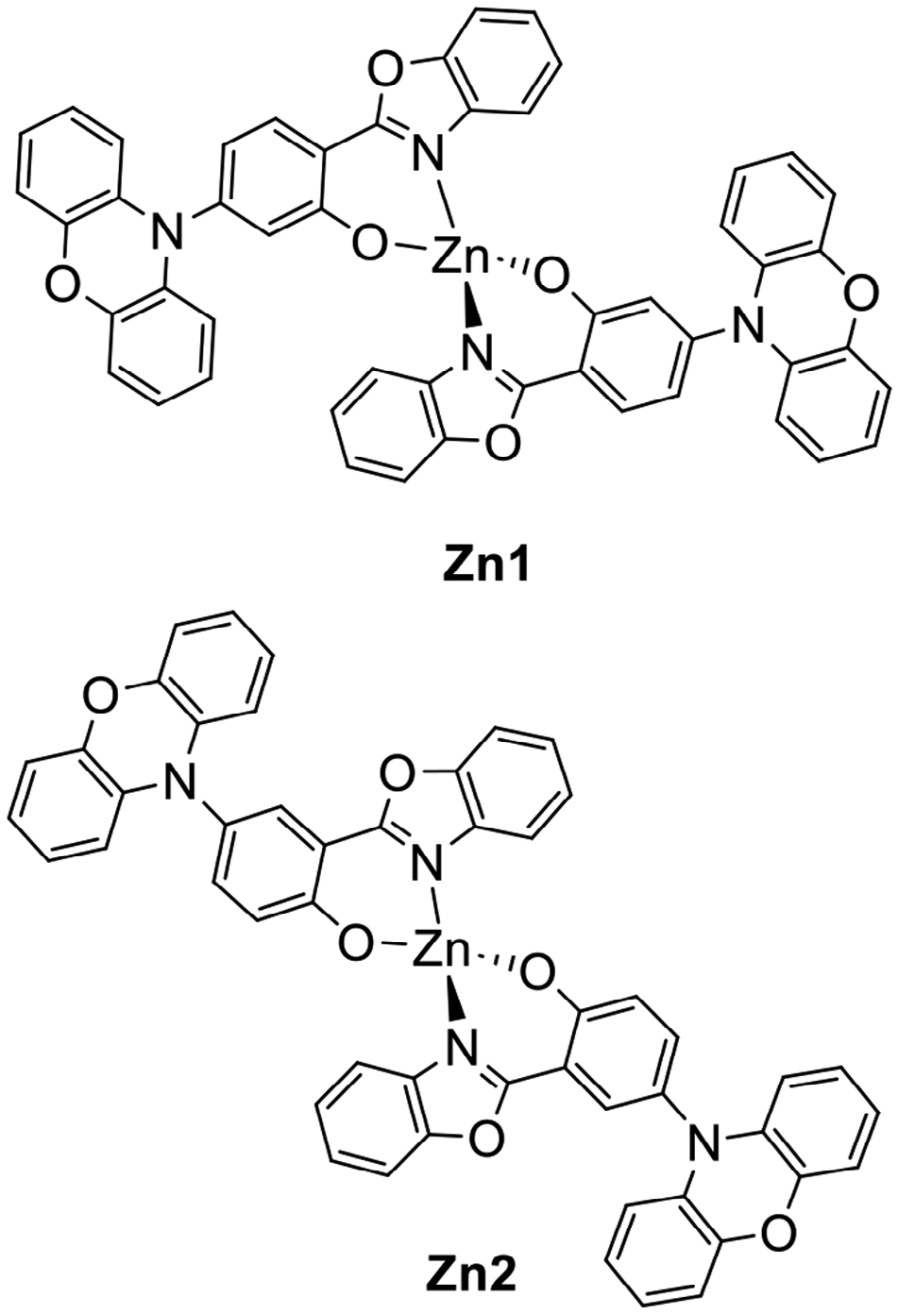
Structures of the first TADF Zn(II) complexes prepared by Adachi and coworkers.

The first Zn(II) complexes exhibiting TADF properties were presented by Adachi et al. Although Zn(II) complexes are known to possess ligand‐to‐ligand charge transfer (LLCT) states, their strategy consisted of developing a negatively charged ligand, which could enhance the emission of the metal complex via intra‐ligand charge transfer (ILCT) character. The metal core was coordinated by two identical electron‐accepting phenylbenzoxazole (BOX‐OH) units, which are linked to an electron‐donating phenoxazine (PX) moiety (see **Figure** **28**). The two homoleptic Zn(II) complexes differ only in the position of the PX fragment, which is in the *para*‐ and *meta*‐position to the BOX for Zn1 and Zn2, respectively. DFT calculations confirmed that the lowest singlet *S*_1_ and triplet *T*_1_ excited states have an ILCT character, and their energy gap is estimated at 118 cm^−1^ (0.01 eV), enabling the TADF process.^[^ ^106^ ^]^

The photophysical properties of **Zn1** and **Zn2** were measured in 6 wt.%‐doped thin films in 3,3‐di(9*H*‐carbazol‐9‐yl)biphenyl (mCPB) as the host matrix. **Zn1** (*λ*_max_ = 542 nm) displays a bathochromic emission compared to **Zn2** (*λ*_max_ = 523 nm), and a higher PLQY, reaching ≈80%. The *k*_RISC_ was determined to be 16.9 × 10^4^ for **Zn1** and 1.4 × 10^4^ for **Zn2**, demonstrating that the TADF process is enhanced when the direction of the ILCT is facilitated by the inductive effect of the PX unit. A comparison with the corresponding methylated ligand showed that the coordination of the Zn(II) is beneficial to lower Δ*E*_ST_, thus leading to a TADF quantum yield of more than 66% in **Zn1**. **Zn1** was investigated for its application in OLEDs with the following structure: ITO/α‐NPD/6±1 wt.% emitter:mCBP/TPBi/LiF/Al (α‐NPD = 4,4′‐bis[*N*‐(1‐naphthyl)‐*N*‐phenylamino]biphenyl; TPBi = 2,2′,2′'‐(1,3,5‐benzinetriyl)‐tris(1‐phenyl‐1‐H‐benzimidazole)). This OLED exhibited a warm green emission at 542 nm, with an EQE of nearly 20%, demonstrating its potential for efficient light emission. Interestingly, the authors investigated the role of the metal center, preparing the corresponding mononuclear Li(I) and Mg(II) and dinuclear Al(III) complexes, coordinating one and two *para*‐PX‐BOX ligands, respectively. The dinuclear Al(III) complex was thermally unstable and could not be employed for the fabrication of an OLED, while electroluminescent devices with the Mg(II) and Li(I) derivatives showed TADF properties, although the PLQY was slightly lower compared to **Zn1**. These observations indicated that the increase in the atomic number passing from Li(I) to Mg(II) and then to Zn(II) promotes an increase in the SOC, albeit small, that enhances both *k*_ISC_ and *k*_RISC_.^[^ ^106^ ^]^

**Figure 29:**
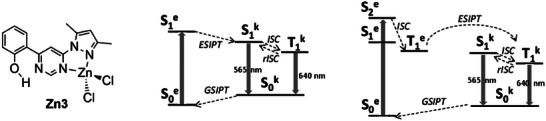
Structure of **Zn3** and possible photophysical processes involving ESIPT and TADF.

Non‐coordinated benzoxazoles are known to exhibit excited‐state intramolecular proton transfer (ESIPT) where the singlet excited state of the enol‐form *S*_1_^e^ is converted into the singlet excited state of the keto‐form *S*_1_^k^, from where the emission is then originated. The significant spatial separation of HOMO and LUMO in ESIPT molecules enables a reduction of the Δ*E*_ST_ and thus TADF. A strategic design of TADF molecules was carried out by Berezin et al. employing a pyrazole‐pyrimidine phenol ligand, which presents ESIPT between the phenol and the vicinal *N*‐atom of the pyrimidine (see **Zn3**, **Figure** **29**). Unexpectedly, the authors found an interplay between TADF and ESIPT by exciting at different wavelengths. While the emission of the free ligand is independent of the excitation wavelength, Zn3 emits at 640 nm with a shoulder at 565 nm, when excited between 240 to 420 nm. With longer excitation wavelengths, the emission intensity at 565 nm increases notably and the signal at 640 nm is covered by the tail of the emission. This study suggests that there is an interplay of singlet and triplet of the enol‐ and the keto‐form, facilitated by the incorporation of the Zn(II) ion. The emission at 640 nm is due to the phosphorescence of the keto‐form (ESIPT) and the emission at 565 nm is promoted by RISC and thus ascribable to TADF (see Figure 29).^[^ ^107^ ^]^

**Figure 30:**
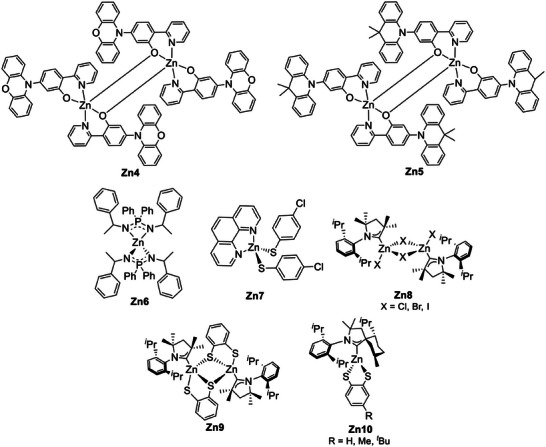
Selected examples of Zn(II) complexes exhibiting TADF.

**Figure 31:**
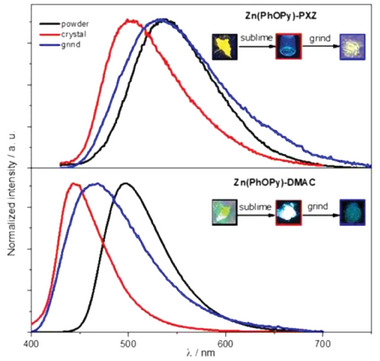
Polymorphism and mechanochromism in **Zn4** and **Zn5**. Reproduced with permission.^[^ ^108^ ^]^ Copyright 2020, Wiley.

A similar strategy to the one employed by Adachi and coworkers^[^ ^106^ ^]^ was developed by Xiong et al. who introduced a donor‐acceptor structure of the ligand to enable the TADF mechanism. The Zn(II) chelating unit is a pyridine‐phenolate (PhOPy), which acts as a donor, and the electron‐acceptor unit is a phenoxazine (PX) or a dimethyldihydroacridine (DMAC). Crystal structural analyses elucidated the molecular structures, which correspond to a dinuclear organization with the oxygen atoms as a bridge between the two Zn(II) nuclei (**Zn4** and **Zn5** in **Figure** **30**), enabling a rigid scaffold that persists in the excited state. These complexes show strong emissions as powders (*λ*_max_ = 538 nm for **Zn4** and *λ*_max_ = 497 for **Zn5**) with quantum yields up to 50%. Interestingly, the PLQY drops to a maximum of 14% for the isolated crystals, although the emission is shifted hypsochromically to 500 and 444 nm respectively for **Zn4** and **Zn5**. Further investigations, supported by DFT and TD‐DFT calculations, showed that RISC is possible with Δ*E*_ST_ equal to 1129 cm^−1^ (0.14 eV) for **Zn4** and only 81 cm^−1^ (0.01 eV) for **Zn5**. Both dinuclear Zn(II) complexes exhibit TADF, besides polymorphism and mechanochromism, as observable in **Figure** **31**.^[^ ^108^ ^]^

Goswami et al. studied the synthesis and photoluminescence properties of the corresponding Zn(II) complexes with iminophosphonamide ligands, in addition to Cu(I) complexes. The homoleptic **Zn6** (see Figure 30) exhibited bright blue‐green phosphorescence at low temperatures (*λ*_max_ = 474 nm). However, at room temperature, the PLQY dropped to 3% and the emission lifetime was only a few ns. Based on the experimental data, the authors estimated Δ*E*_ST_ = 100 cm^−1^ (0.01 eV) supporting the TADF mechanism.^[^ ^44^ ^]^

The Zn(II) phenanthroline bis‐thiolate complex **Zn7** (see Figure 30) was first prepared by Crosby and coworkers, who suggested a dual emission coming from non‐equilibrated ^1^LLCT and ^3^LLCT excited states.^[^ ^109^ ^]^ This derivative was further investigated by Marian, Steffen, and coworkers to elucidate the possibility of RISC and, thus, TADF. Temperature‐dependent emission spectra in the solid state show that the ^3^LLCT state is the main emissive excited state with a broad emission centered at 587 nm, while there are two less intense emissions at higher energies originating from ^1^LC and ^3^LC states localized on the phenanthroline. This was rationalized as originating from the free rotation of the aryl‐thiolate ligands, forming two confirmers in solution. In the solid state, these rotations are highly hindered, thus the internal conversion between the LC and LLCT states becomes less probable. Moreover, looking at the behavior of the excited state lifetime, it was noticed that below 23 K, a sigmoidal fitting could describe the trend of the average *τ*, while above that temperature a rapid decrease below 0.5 µs occurs. This could only be explained if the ^1^LLCT and ^3^LLCT states were in energetic proximity and an equilibrium could be established, even at low temperatures such as 77 K.^[^ ^110^ ^]^ Further mechanistic and experimental observations led to the conclusion that internal conversion populates ^1^LLCT and ^3^LLCT from the singlet and triplet LC states of the phenanthroline, as Crosby already suggested.^[^ ^109^ ^]^ Nevertheless, these two states have Δ*E*_ST_ ≈ 423 cm^−1^ (0.052 eV) enabling TADF.^[^ ^110^ ^]^

**Figure 32:**
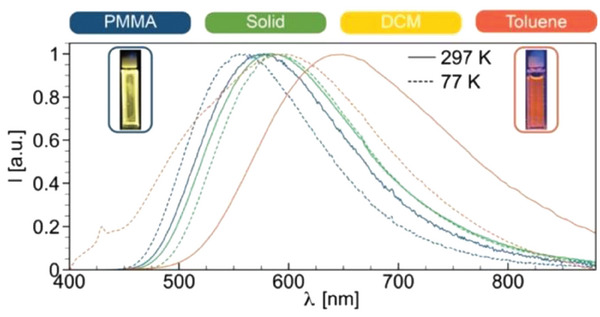
Emission spectra of **Zn9** in different media. Reproduced with permission.^[^ ^112^ ^]^ Copyright 2023, Wiley.

In analogy with linear NHC‐Cu complexes, the development of Zn(II) complexes using the strong *σ*‐donating and π‐accepting cAACs as ligands was pursued by Marian, Steffen et al. The butterfly‐type dimeric structures **Zn8** (see Figure 30) were prepared with Cl, Br and I as bridging halides when THF was used in the synthesis, whereas in a coordinating solvent such as acetonitrile, the corresponding mononuclear complexes were formed. The three dimers show broad dual emissions due to ^3^XCT/LE excited states, but the intensity ratio of the bands changed over short times due to the instability of Zn‐X bonds at room temperature.^[^ ^111^ ^]^ More recently, an improved structural design was afforded by the same groups employing the same cAAC and different chelating dianionic phenyldithiolates. The corresponding binuclear complex **Zn9** (see Figure 30) exhibited noticeable emissions between 577 and 657 nm in the solid state, solution, and PMMA matrices ascribed to ^1/3^LL/LMCT (see **Figure** **32**). Fast TADF was supported by experimental data and DFT calculations, with the Δ*E*_ST_ estimated at 637 cm^−1^ (0.08 eV) and *k*_r_ = 1.5 × 10^5^ s^−1^ at room temperature. The visible light absorption properties and lifetimes in the hundreds of ns range in solution make **Zn9** a suitable candidate to be applied in Dexter energy transfer photocatalysis.^[^ ^112^ ^]^ Steffen and coworkers reported the mononuclear carbene‐dithiolate **Zn10** (see Figure 30) which was characterized by one of the highest *k*_r_ reported so far for Zn(II) complexes and equal to 1.2 × 10^6^ s^−1^ at 297 K. In this case, DFT/MRCI (multireference configuration interaction) calculations highlighted that TADF is favored by a dihedral angle between the two ligands in the 36–40° range, which determines an efficient RISC, and results in an Δ*E*_ST_ of 452 cm^−1^ (0.06 eV).^[^ ^113^ ^]^

### 4.6 Group 14: Sn(IV) complexes

**Figure 33:**
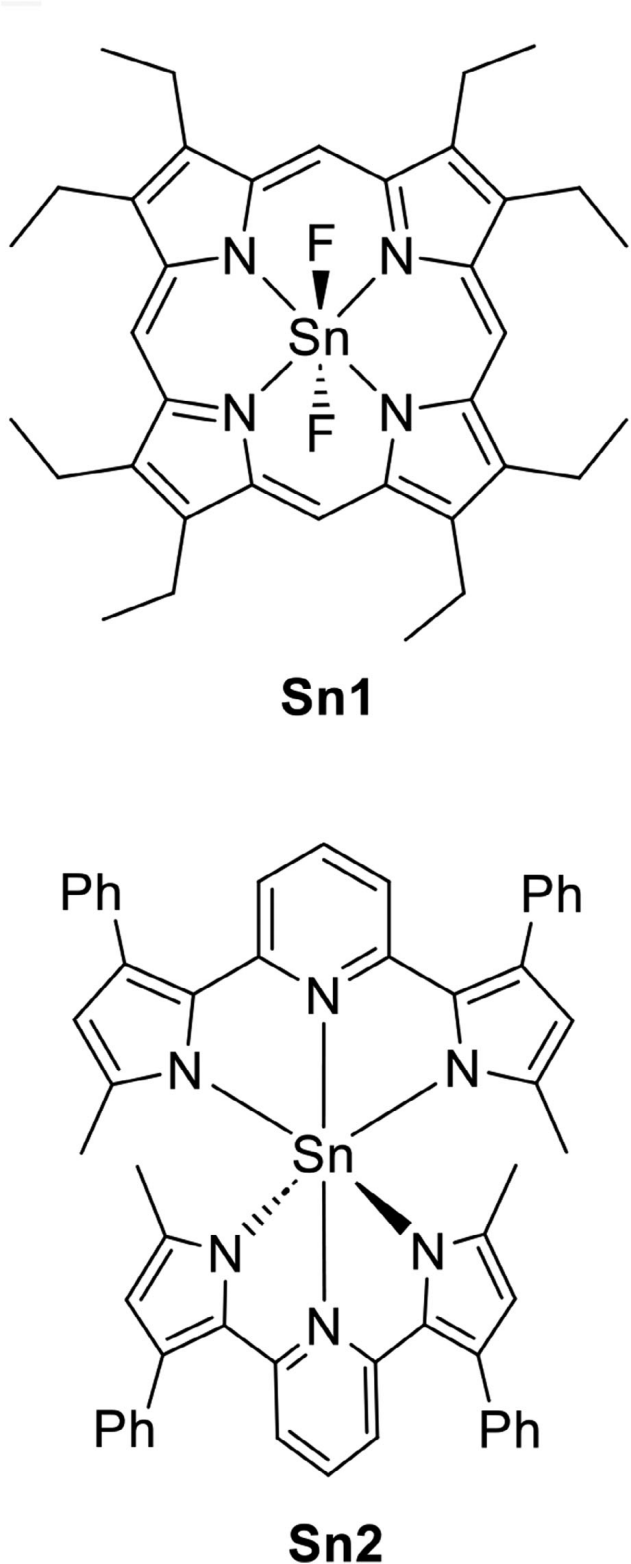
Structures of the TADF Sn(IV) complexes.

Despite not being a transition metal element and being relatively scarce on the earth's crust, Sn is more abundant than Ag (2 ppm vs. 0.08 ppm concentration). The Sn(IV) porphyrin **Sn1** shown in **Figure** **33** was the first compound exhibiting TADF that was successfully tested in an OLED by Adachi and coworkers. A thin film of poly(vinylcarbazole) doped with 2 wt.% of **Sn1** was used to study PF and DF. The phosphorescence could be detected only below 200 K and an intense TADF emission centered at 570 nm could be observed above 400 K. The OLED structure was built on top of an ITO/PEDOT anode and a layer of MgAg was deposited before the Ag cathode, acting as an electron injection layer. Although this structure was not further optimized and the onset of the current injection was 10 V, the electroluminescence showed a strong TADF emission at 400 K. The estimated energy gap Δ*E*_ST_ was 1935 cm^−1^ (0.24 eV), which is why an efficient RISC could be promoted only at higher temperatures. Nevertheless, although this result was not exceptional, this pivotal investigation set the basis for the use of TADF compounds in OLEDs.^[^ ^114^ ^]^

Recently, Milsmann and Castellano developed **Sn2**, a homoleptic hexacoordinated Sn(IV) complex with bis(pyrazole) pyridine‐derived ligands. Due to the presence of a heavy atom and large SOC, Sn(IV) complexes usually show phosphorescent emission. In line with this, the emission in THF solution (*λ*_max_ 520 nm) presented an unsymmetrical profile with a PLQY of 32% in the absence of oxygen, while in aerated solution the formation of ^1^O_2_ could be detected. In 2‐methyl THF glass matrix, Sn2 exhibited dual emission: a low energy band with a maximum of 580 nm appears, while the band at 520 nm decreases in intensity. These findings support the hypothesis of an equilibrium between the *T*_1_ and S_1_ states when increasing the temperature. The Δ*E*_ST_ estimated by the measurements at low temperatures is higher compared to other TADF compounds, being 2524 cm^−1^ (0.31 eV). With the support of DFT calculations, the first singlet *S*_1_ was assigned to an LLCT state while the first triplet state *T*_1_ had an LC nature. Thus, the orthogonality of the two ligands favors the charge‐transfer character of the excited state, generating a set of LUMO with similar energy.^[^ ^115^ ^]^

### 4.7 Conclusion and Perspectives

In this review, we provided several examples of earth‐abundant metal complexes that exhibit efficient TADF and are competitive with commercially available Ir(III) and Pt(II) complexes due to a combination between small energy gaps Δ*E*_ST_ and high *S*_1_ → *S*_0_ oscillator strengths, and their exploitation in photoconversion devices, such as OLEDs and LECs. In addition, TADF properties are highly desirable since the weak SOC of some earth‐abundant metals is enough to enable the RISC, preventing an inefficient emission from the electronically populated triplet emitting states and thus their use as phosphorescent emitters.

The versatility of TMCs which can potentially be prepared depending on the metal center and the ligands, and the variety of possible emission mechanisms enable their use in a wide range of applications beyond OLEDs and LECs, including X‐ray detection, photocatalysis, and sensing. As illustrated by the number of examples described herein, particular attention was devoted to Cu(I) complexes as a possible replacement for rare and expensive second‐ and third‐row transition metal elements. Its d^10^ configuration and the absence of possible *d*‐*d* transitions make it particularly attractive for such purposes, in combination with a moderately strong SOC effect and the tunability provided by the MLCT mechanism. As both the ligands and the metal center are directly involved in the emission mechanism, the photoluminescence properties can be easily adjusted in terms of maxima, lifetimes, and quantum yields.

The choice of earth‐abundant metal‐based TADF emitters in lighting and display technologies presents a promising alternative to traditional phosphorescent noble‐metal‐based emitters. These more readily available and cost‐effective metals not only reduce the overall costs of production and enhance the sustainability of the devices but also contribute to an increased radiative decay from the singlet excited state, contributing to the achievement of an ideal internal quantum efficiency of 1. Moreover, operational stabilities and device lifetimes are fairly competing with those of OLEDs based on Ir(III) or Pt(II) complexes, for example, the device based on **Cu44** (EQE: 23.6% and device lifetime: 1300 h, vide supra). Moreover, earth‐abundant metal‐based TADF complexes present an advantage also compared to the pure organic TADF emitters, since they usually exhibit shorter emission lifetimes, facilitating the operational conditions of devices.

In conclusion, the current quest for sustainability is likely to lead to the development of increasingly efficient TADF transition metal complexes and thus their application as photoconversion materials combining processability, stability, and structural diversity. We believe that the overview of the examples here presented will help in the design of complexes with improved TADF properties to be applied for such purposes, together with the development of new architectures for efficient and stable OLEDs and LECs, to drive the advancement of more environmentally benign next generation lightening and display technologies.

## Conflict of Interest

The authors declare no conflict of interest.

## References

[1] M. Y. Wong , E. Zysman‐Colman , Adv. Mater. 2017, 29, 1605444. 10.1002/adma.201605444 28256751

[2] a) O. S. Wenger , J. Am. Chem. Soc. 2018, 140, 13522; 30351136 10.1021/jacs.8b08822

[3] G. U. Mahoro , J. Fernandez‐Cestau , J. L. Renaud , P. B. Coto , R. D. Costa , S. Gaillard , Adv. Opt. Mater. 2020, 8, 2000260.

[4] a) C. A. Parker , C. G. Hatchard , Trans. Faraday Society 1961, 57, 1894;

[5] G. Blasse , D. R. McMillin , Chem. Phys. Lett. 1980, 70, 1.

[6] A. Steffen , B. Hupp , in Comprehensive Coordination Chemistry III (Eds.: E. C. Constable , G. Parkin , L. Q. Jr. ), Elsevier, Netherlands 2021, 466.

[7] C. Bizzarri , F. Hundemer , J. Busch , S. Bräse , Polyhedron 2018, 140, 51.

[8] Z. Han , X. Y. Dong , S. Q. Zang , Adv. Opt. Mater. 2021, 9, 2100081.

[9] H. Yersin , R. Czerwieniec , M. Z. Shafikov , A. F. Suleymanova , ChemPhysChem 2017, 18, 3508. 29083512 10.1002/cphc.201700872

[10] C. E. Housecroft , E. C. Constable , J. Mater. Chem. C 2022, 10, 4456. 10.1039/d1tc04028f PMC8944257 35433007

[11] M. J. Leitl , F. R. Küchle , H. A. Mayer , L. Wesemann , H. Yersin , J. Phys. Chem. A 2013, 117, 11823. 23967801 10.1021/jp402975d

[12] N. Holonyak , S. F. Bevacqua , Appl. Phys. Lett. 1962, 1, 82.

[13] a) G. Hong , X. Gan , C. Leonhardt , Z. Zhang , J. Seibert , J. M. Busch , S. Bräse , Adv. Mater. 2021, 33, 2005630; 10.1002/adma.202005630 33458866

[14] D. Wang , J. Hauptmann , C. May , MRS Advances 2019, 4, 1367.

[15] X. Xu , Y. Zhao , Y. Liu , Small 2023, 19, 2206309. 10.1002/smll.202206309 36794301

[16] a) T. Wang , Y. Z. Wang , L. C. Jing , Q. Zhu , A. S. Ethiraj , W. Geng , Y. Tian , Z. Zhu , Z. Meng , H. Z. Geng , Carbon 2021, 172, 379;

[17] N. Thejo Kalyani , S. J. Dhoble , Renewable Sustainable Energy Rev. 2015, 44, 319.

[18] a) Q. Pei , G. Yu , C. Zhang , Y. Yang , A. J. Heeger , Science 1995, 269, 1086; 17755530 10.1126/science.269.5227.1086

[19] K. Schlingman , Y. Chen , R. S. Carmichael , T. B. Carmichael , Adv. Mater. 2021, 33, 2006863. 10.1002/adma.202006863 33852176

[20] H. Uoyama , K. Goushi , K. Shizu , H. Nomura , C. Adachi , Nature 2012, 492, 234. 23235877 10.1038/nature11687

[21] H. Mieno , R. Kabe , M. D. Allendorf , C. Adachi , Chem. Commun. 2018, 54, 631. 10.1039/c7cc08595h 29299562

[22] Y. Zhang , T. S. Lee , J. M. Favale , D. C. Leary , J. L. Petersen , G. D. Scholes , F. N. Castellano , C. Milsmann , Nat. Chem. 2020, 12, 345. 32203439 10.1038/s41557-020-0430-7

[23] J. Zhang , J. Ma , S. Zhang , X. Lou , Y. Ding , Y. Li , M. Xu , X. Xie , X. Jiao , X. Dou , X. Wang , B. Tang , ACS Nano 2023, 17, 23430. 38011322 10.1021/acsnano.3c05106

[24] A. Russegger , A. C. Debruyne , D. C. Berrio , S. Fuchs , J. Marzi , K. Schenke‐Layland , R. I. Dmitriev , S. M. Borisov , Adv. Opt. Mater. 2023, 11, 2202720.

[25] K. T. Yeung , W. P. To , C. Sun , G. Cheng , C. Ma , G. S. M. Tong , C. Yang , C. M. Che , Angew. Chem., Int. Ed. 2017, 56, 133. 10.1002/anie.201608240 27918133

[26] K. T. Chan , T. L. Lam , D. Yu , L. Du , D. L. Phillips , C. L. Kwong , G. S. M. Tong , G. Cheng , C. M. Che , Angew. Chem., Int. Ed. 2019, 58, 14896. 10.1002/anie.201906698 31321857

[27] D. Yu , W. P. To , G. S. M. Tong , L. L. Wu , K. T. Chan , L. Du , D. L. Phillips , Y. Liu , C. M. Che , Chem. Sci. 2020, 11, 6370. 32874518 10.1039/d0sc01340d PMC7448528

[28] M. T. Buckner , D. R. McMillin , J. Chem. Soc., Chem. Commun. 1978, 10.1039/C39780000759.

[29] Y. Liu , S. C. Yiu , C. L. Ho , W. Y. Wong , Coord. Chem. Rev. 2018, 375, 514.

[30] a) J. Beaudelot , S. Oger , S. Perusko , T. A. Phan , T. Teunens , C. Moucheron , G. Evano , Chem. Rev. 2022, 122, 16365; 36350324 10.1021/acs.chemrev.2c00033

[31] a) H. Yersin , Highly Efficient OLEDs: Materials Based on Thermally Activated Delayed Fluorescence, Wiley‐VCH, Weinheim, Germany 2019;

[32] M. Wallesch , D. Volz , D. M. Zink , U. Schepers , M. Nieger , T. Baumann , S. Bräse , Chem. ‐ Eur. J. 2014, 20, 6578. 24757123 10.1002/chem.201402060

[33] D. M. Zink , M. Bachle , T. Baumann , M. Nieger , M. Kuhn , C. Wang , W. Klopper , U. Monkowius , T. Hofbeck , H. Yersin , S. Brase , Inorg. Chem. 2013, 52, 2292. 23061380 10.1021/ic300979c

[34] T. Hofbeck , T. A. Niehaus , M. Fleck , U. Monkowius , H. Yersin , Molecules 2021, 26, 3415. 34200044 10.3390/molecules26113415 PMC8200198

[35] A. Schinabeck , M. J. Leitl , H. Yersin , J. Phys. Chem. Lett. 2018, 9, 2848. 29750529 10.1021/acs.jpclett.8b00957

[36] a) J. M. Busch , D. M. Zink , P. Di Martino‐Fumo , F. R. Rehak , P. Boden , S. Steiger , O. Fuhr , M. Nieger , W. Klopper , M. Gerhards , S. Bräse , Dalton Trans. 2019, 48, 15687; 31524902 10.1039/c9dt02447f

[37] J. M. Busch , D. S. Koshelev , A. A. Vashchenko , O. Fuhr , M. Nieger , V. V. Utochnikova , S. Bräse , Inorg. Chem. 2021, 60, 2315. 33464050 10.1021/acs.inorgchem.0c03187

[38] T. Hofbeck , U. Monkowius , H. Yersin , J. Am. Chem. Soc. 2015, 137, 399. 25486064 10.1021/ja5109672

[39] A. V. Artem'ev , M. P. Davydova , A. S. Berezin , M. R. Ryzhikov , D. G. Samsonenko , Inorg. Chem. 2020, 59, 10699. 32687333 10.1021/acs.inorgchem.0c01171

[40] M. P. Davydova , A. S. Berezin , D. G. Samsonenko , A. V. Artem'ev , Inorg. Chim. Acta 2021, 521, 120347.

[41] a) A. Kobayashi , T. Hasegawa , M. Yoshida , M. Kato , Inorg. Chem. 2016, 55, 1978. 26866384 10.1021/acs.inorgchem.5b02160

[42] A. Y. Baranov , A. S. Berezin , D. G. Samsonenko , A. S. Mazur , P. M. Tolstoy , V. F. Plyusnin , I. E. Kolesnikov , A. V. Artem'ev , Dalton Trans. 2020, 49, 3155. 32083636 10.1039/d0dt00192a

[43] Y. V. Demyanov , E. H. Sadykov , M. I. Rakhmanova , A. S. Novikov , I. Y. Bagryanskaya , A. V. Artem'ev , Molecules 2022, 27, 6059. 36144790 10.3390/molecules27186059 PMC9503387

[44] B. Goswami , T. J. Feuerstein , R. Yadav , S. Lebedkin , P. J. Boden , S. T. Steiger , G. Niedne‐Schatteburg , M. Gerhards , M. M. Kappes , P. W. Roesky , Chem. ‐ Eur. J. 2021, 27, 15110. 10.1002/chem.202101247 PMC8596734 33899967

[45] M. Klein , N. Rau , M. Wende , J. Sundermeyer , G. Cheng , C. M. Che , A. Schinabeck , H. Yersin , Chem. Mater. 2020, 32, 10365.

[46] J. C. Deaton , S. C. Switalski , D. Y. Kondakov , R. H. Young , T. D. Pawlik , D. J. Giesen , S. B. Harkins , A. J. M. Miller , S. F. Mickenberg , J. C. Peters , J. Am. Chem. Soc. 2010, 132, 9499. 20557045 10.1021/ja1004575

[47] C. L. Linfoot , M. J. Leitl , P. Richardson , A. F. Rausch , O. Chepelin , F. J. White , H. Yersin , N. Robertson , Inorg. Chem. 2014, 53, 10854. 25054425 10.1021/ic500889s

[48] A. Lavie‐Cambot , M. Cantuel , Y. Leydet , G. Jonusauskas , D. M. Bassani , N. D. McClenaghan , Coord. Chem. Rev. 2008, 252, 2572.

[49] a) C. Femoni , S. Muzzioli , A. Palazzi , S. Stagni , S. Zacchini , F. Monti , G. Accorsi , M. Bolognesi , N. Armaroli , M. Massi , G. Valenti , M. Marcaccio , Dalton Trans. 2013, 42, 997; 23108182 10.1039/c2dt32056h

[50] E. Leoni , J. Mohanraj , M. Holler , M. Mohankumar , I. Nierengarten , F. Monti , A. Sournia‐Saquet , B. Delavaux‐Nicot , J. F. I. Nierengarten , N. Armaroli , Inorg. Chem. 2018, 57, 15537. 30481016 10.1021/acs.inorgchem.8b02879

[51] Q. Zhang , Q. Zhou , Y. Cheng , L. Wang , D. Ma , X. Jing , F. Wang , Adv. Mater. 2004, 16, 432.

[52] Q. Zhang , Q. Zhou , Y. Cheng , L. Wang , D. Ma , X. Jing , F. Wang , Adv. Funct. Mater. 2006, 16, 1203.

[53] R. Czerwieniec , K. Kowalski , H. Yersin , Dalton Trans. 2013, 42, 9826. 23722734 10.1039/c3dt51006a

[54] C. Li , C. F. R. Mackenzie , S. A. Said , A. K. Pal , M. A. Haghighatbin , A. Babaei , M. Sessolo , D. B. Cordes , A. M. Z. Slawin , P. C. J. Kamer , H. J. Bolink , C. F. Hogan , E. Zysman‐Colman , Inorg. Chem. 2021, 60, 10323. 34197094 10.1021/acs.inorgchem.1c00804

[55] M. D. Weber , M. Viciano‐Chumillas , D. Armentano , J. Cano , R. D. Costa , Dalton Trans. 2017, 46, 6312. 28452386 10.1039/c7dt00810d

[56] S. Keller , F. Brunner , J. M. Junquera‐Hernández , A. Pertegás , M. G. La‐Placa , A. Prescimone , E. C. Constable , H. J. Bolink , E. Ortí , C. E. Housecroft , ChemPlusChem 2018, 83, 217. 31957280 10.1002/cplu.201700501

[57] S. Keller , A. Prescimone , M. G. La Placa , J. M. Junquera‐Hernández , H. J. Bolink , E. C. Constable , M. Sessolo , E. Ortí , C. E. Housecroft , RSC Adv. 2020, 10, 22631. 35514545 10.1039/d0ra03824e PMC9054616

[58] a) M. Bouzrati‐Zerelli , N. Guillaume , F. Goubard , T. T. Bui , S. Villotte , C. Dietlin , F. Morlet‐Savary , D. Gigmes , J. P. Fouassier , F. Dumur , J. Lalevée , New J. Chem. 2018, 42, 8261;

[59] X. L. Chen , R. Yu , Q. K. Zhang , L. J. Zhou , X. Y. Wu , Q. Zhang , C. Z. Lu , Chem. Mater. 2013, 25, 3910.

[60] X. W. Chen , L. H. He , P. Ju , J. L. Chen , S. J. Liu , H. R. Wen , J. Mater. Chem. C 2020, 8, 16160.

[61] L. M. Cavinato , S. Wölfl , A. Pöthig , E. Fresta , C. Garino , J. Fernandez‐Cestau , C. Barolo , R. D. Costa , Adv. Mater. 2022, 34, 2109228. 10.1002/adma.202109228 35034407

[62] a) A. N. Gusev , E. Braga , E. Zamnius , M. A. Kiskin , A. Ali , G. Baryshnikov , W. Linert , Dalton Trans. 2023, 52, 14995; 37811719 10.1039/d3dt02633g

[63] G. U. Mahoro , E. Fresta , M. Elie , D. Di Nasso , Q. Zhang , J. F. Lohier , J. L. Renaud , M. Linares , R. Wannemacher , J. Cabanillas‐Gonzalez , R. D. Costa , S. Gaillard , Dalton Trans. 2021, 50, 11049. 34286773 10.1039/d1dt01689j

[64] G. Giobbio , L. M. Cavinato , E. Fresta , A. Montrieul , G. Umuhire Mahoro , J. F. Lohier , J. L. Renaud , M. Linares , S. Gaillard , R. D. Costa , Adv. Funct. Mater. 2023, 33, 2304668.

[65] F. Zhang , Y. Guan , X. Chen , S. Wang , D. Liang , Y. Feng , S. Chen , S. Li , Z. Li , F. Zhang , C. Lu , G. Cao , B. Zhai , Inorg. Chem. 2017, 56, 3742. 28304161 10.1021/acs.inorgchem.6b01847

[66] L. Lin , D. H. Chen , R. Yu , X. L. Chen , W. J. Zhu , D. Liang , J. F. Chang , Q. Zhang , C. Z. Lu , J. Mater. Chem. C 2017, 5, 4495.

[67] R. Czerwieniec , J. Yu , H. Yersin , Inorg. Chem. 2011, 50, 8293. 21812428 10.1021/ic200811a

[68] L. Bergmann , G. J. Hedley , T. Baumann , S. Bräse , I. D. W. Samuel , Sci. Adv. 2016, 2, e150088. 10.1126/sciadv.1500889 PMC4705038 26767194

[69] X. W. Chen , H. L. Yuan , L. H. He , J. L. Chen , S. J. Liu , H. R. Wen , G. Zhou , J. Y. Wang , W. Y. Wong , Inorg. Chem. 2019, 58, 14478. 31618013 10.1021/acs.inorgchem.9b01972

[70] A. Alconchel , O. Crespo , P. García‐Orduña , M. C. Gimeno , Inorg. Chem. 2021, 60, 18521. 34812617 10.1021/acs.inorgchem.1c03092 PMC8653344

[71] a) Z. C. Su , C. C. Zheng , G. Cheng , C. M. Che , S. J. Xu , J. Mater. Chem. C 2017, 5, 4488;

[72] A. Alconchel , O. Crespo , M. C. Gimeno , Inorg. Chem. 2023, 62, 10431. 37348058 10.1021/acs.inorgchem.3c01409 PMC10324396

[73] A. Muthig , T. Martin , O. Mrózek , T. Ferschke , M. Rödel , B. Ewald , J. Kuhnt , C. Lenczyk , J. Pflaum , A. Steffen , J. Am. Chem. Soc. 2023, 145, 4438. 36795037 10.1021/jacs.2c09458

[74] H. Amouri , Chem. Rev. 2023, 123, 230. 36315851 10.1021/acs.chemrev.2c00206

[75] M. J. Leitl , V. A. Krylova , P. I. Djurovich , M. E. Thompson , H. Yersin , J. Am. Chem. Soc. 2014, 136, 16032. 25260042 10.1021/ja508155x

[76] M. Elie , F. Sguerra , F. Di Meo , M. D. Weber , R. Marion , A. Grimault , J. F. Lohier , A. Stallivieri , A. Brosseau , R. B. Pansu , J. L. Renaud , M. Linares , M. Hamel , R. D. Costa , S. Gaillard , ACS Appl. Mater. Interfaces 2016, 8, 14678. 27224961 10.1021/acsami.6b04647

[77] M. D. Weber , E. Fresta , M. Elie , M. E. Miehlich , J. L. Renaud , K. Meyer , S. Gaillard , R. D. Costa , Adv. Funct. Mater. 2018, 28, 1707423.

[78] M. Elie , M. D. Weber , F. Di Meo , F. Sguerra , J. F. Lohier , R. B. Pansu , J. L. Renaud , M. Hamel , M. Linares , R. D. Costa , S. Gaillard , Chem. ‐ Eur. J. 2017, 23, 16328. 28872717 10.1002/chem.201703270

[79] A. Liske , L. Wallbaum , T. Hölzel , J. Föller , M. Gernert , B. Hupp , C. Ganter , C. M. Marian , A. Steffen , Inorg. Chem. 2019, 58, 5433. 31002248 10.1021/acs.inorgchem.9b00337

[80] A. Ying , S. Gong , Chem. ‐ Eur. J. 2023, 29, e202301885. 37431981 10.1002/chem.202301885

[81] J. Li , L. Wang , Z. Zhao , X. Li , X. Yu , P. Huo , Q. Jin , Z. Liu , Z. Bian , C. Huang , Angew. Chem., Int. Ed. 2020, 59, 8210. 10.1002/anie.201916379 31985121

[82] A. Ruduss , B. Turovska , S. Belyakov , K. A. Stucere , A. Vembris , K. Traskovskis , Inorg. Chem. 2022, 61, 2174. 35038860 10.1021/acs.inorgchem.1c03371

[83] H. J. Wang , Y. Liu , B. Yu , S. Q. Song , Y. X. Zheng , K. Liu , P. Chen , H. Wang , J. Jiang , T. Y. Li , Angew. Chem., Int. Ed. 2023, 62, 202217195. 10.1002/anie.202217195 36542446

[84] M. Gernert , L. Balles‐Wolf , F. Kerner , U. Müller , A. Schmiedel , M. Holzapfel , C. M. Marian , J. Pflaum , C. Lambert , A. Steffen , J. Am. Chem. Soc. 2020, 142, 8897. 32302135 10.1021/jacs.0c02234

[85] R. Tang , S. Xu , T. L. Lam , G. Cheng , L. Du , Q. Wan , J. Yang , F. F. Hung , K. H. Low , D. L. Phillips , C. M. Che , Angew. Chem. 2022, 134, e202203982. 10.1002/anie.202203982 35647660

[86] A. Ying , Y. Ai , C. Yang , S. Gong , Angew. Chem., Int. Ed. 2022, 61, e202210490. 10.1002/anie.202210490 36106609

[87] A. Ying , Y. H. Huang , C. H. Lu , Z. Chen , W. K. Lee , X. Zeng , T. Chen , X. Cao , C. C. Wu , S. Gong , C. Yang , ACS Appl. Mater. Interfaces 2021, 13, 13478. 33689279 10.1021/acsami.0c22109

[88] A. M. T. Muthig , J. Wieland , C. Lenczyk , S. Koop , J. Tessarolo , G. H. Clever , B. Hupp , A. Steffen , Chem. ‐ Eur. J. 2023, 29, e202300946. 37272620 10.1002/chem.202300946

[89] H. Ohara , A. Kobayashi , M. Kato , Dalton Trans. 2014, 43, 17317. 25315634 10.1039/c4dt02709d

[90] A. Gusev , M. Kiskin , E. Braga , E. Zamnius , M. Kryukova , N. Karaush‐Karmazin , G. Baryshnikov , B. Minaev , W. Linert , RSC Adv. 2023, 13, 3899. 36756544 10.1039/d2ra06986e PMC9890518

[91] S. Liu , J. Y. Zhang , C. M. Liu , G. J. Yin , M. Wu , C. X. Du , B. Zhang , Polyhedron 2022, 218, 115761.

[92] A. V. Artem'ev , A. Y. Baranov , A. S. Berezin , D. V. Stass , C. Hettstedt , U. Y. A. Kuzmina , K. Karaghiosoff , I. Y. Bagryanskaya , Int. J. Mol. Sci. 2023, 24, 5145. 36982219 10.3390/ijms24065145 PMC10049412

[93] M. Osawa , Chem. Commun. 2014, 50, 1801. 10.1039/c3cc47871h 24396864

[94] M. Osawa , I. Kawata , R. Ishii , S. Igawa , M. Hashimoto , M. Hoshino , J. Mater. Chem. C 2013, 1, 4375.

[95] A. V. Artem'ev , E. P. Doronina , M. I. Rakhmanova , O. A. Tarasova , I. Y. Bagryanskaya , N. A. Nedolya , Inorg. Chem. Front. 2019, 6, 671.

[96] a) T. Gneuß , M. J. Leitl , L. H. Finger , H. Yersin , J. Sundermeyer , Dalton Trans. 2015, 44, 20045; 26525145 10.1039/c5dt03065j

[97] M. Z. Shafikov , A. F. Suleymanova , R. Czerwieniec , H. Yersin , Chem. Mater. 2017, 29, 1708. 10.1021/acs.inorgchem.7b02002 29053269

[98] X. M. Gan , R. Yu , X. L. Chen , M. Yang , L. Lin , X. Y. Wu , C. Z. Lu , Dalton Trans. 2018, 47, 5956. 29666869 10.1039/c8dt00837j

[99] A. V. Artem'ev , M. Z. Shafikov , A. Schinabeck , O. V. Antonova , A. S. Berezin , I. Y. Bagryanskaya , P. E. Plusnin , H. Yersin , Inorg. Che. Front. 2019, 6, 3168.

[100] J. M. Busch , F. R. Rehak , V. Ferraro , M. Nieger , M. Kemell , O. Fuhr , W. Klopper , S. Brase , ACS Omega 2024, 9, 2220. 38250424 10.1021/acsomega.3c05755 PMC10795044

[101] R. Hamze , S. Shi , S. C. Kapper , D. S. Muthiah Ravinson , L. Estergreen , M. C. Jung , A. C. Tadle , R. Haiges , P. I. Djurovich , J. L. Peltier , R. Jazzar , G. Bertrand , S. E. Bradforth , M. E. Thompson , J. Am. Chem. Soc. 2019, 141, 8616. 31062972 10.1021/jacs.9b03657

[102] E. Fresta , J. M. Carbonell‐Vilar , J. Yu , D. Armentano , J. Cano , M. Viciano‐Chumillas , R. D. Costa , Adv. Funct. Mater. 2019, 29, 1901797. 10.1039/c9dt00772e 31011739

[103] W. F. Wang , M. J. Xie , P. K. Wang , J. Lu , B. Y. Li , M. S. Wang , S. H. Wang , F. K. Zheng , G. C. Guo , Angew. Chem., Int. Ed. 2024, 63, e202318026. 10.1002/anie.202318026 38157447

[104] N. Lüdtke , A. Steffen , C. M. Marian , Inorg. Chem. 2022, 61, 20896. 36490354 10.1021/acs.inorgchem.2c03301

[105] J. A. Kübler , B. Pfund , O. S. Wenger , JACS Au 2022, 2, 2367. 36311829 10.1021/jacsau.2c00442 PMC9597861

[106] Y. Sakai , Y. Sagara , H. Nomura , N. Nakamura , Y. Suzuki , H. Miyazaki , C. Adachi , Chem. Commun. 2015, 51, 3181. 10.1039/c4cc09403d 25605520

[107] A. S. Berezin , K. A. Vinogradova , V. P. Krivopalov , E. B. Nikolaenkova , V. F. Plyusnin , A. S. Kupryakov , N. V. Pervukhina , D. Y. Naumov , M. B. Bushuev , Chem. ‐ Eur. J. 2018, 24, 12790. 29939444 10.1002/chem.201802876

[108] J. Xiong , K. Li , T. Teng , X. Chang , Y. Wei , C. Wu , C. Yang , Chem. ‐ Eur. J. 2020, 26, 6887. 32162737 10.1002/chem.202000572

[109] a) R. G. Highland , G. A. Crosby , Chem. Phys. Lett. 1985, 119, 454;

[110] N. Lüdtke , J. Kuhnt , T. Heil , A. Steffen , C. M. Marian , ChemPhotoChem 2023, 7, 202200142.

[111] O. Mrózek , M. Gernert , A. Belyaev , M. Mitra , L. Janiak , C. M. Marian , A. Steffen , Chem. ‐ Eur. J. 2022, 28, e202201114. 35583397 10.1002/chem.202201114 PMC9544448

[112] O. Mrózek , M. Mitra , B. Hupp , A. Belyaev , N. Ludtke , D. Wagner , C. Wang , O. S. Wenger , C. M. Marian , A. Steffen , Chem. ‐ Eur. J. 2023, 29, e202203980. 36637038 10.1002/chem.202203980

[113] M. Mitra , O. Mrózek , M. Putscher , J. Guhl , B. Hupp , A. Belyaev , C. M. Marian , A. Steffen , Angew. Chem., Int. Ed. 2024, 63, 202316300. 10.1002/anie.202316300 38063260

[114] A. Endo , M. Ogasawara , A. Takahashi , D. Yokoyama , Y. Kato , C. Adachi , Adv. Mater. 2009, 21, 4802. 21049498 10.1002/adma.200900983

[115] A. S. Gowda , T. S. Lee , M. C. Rosko , J. L. Petersen , F. N. Castellano , C. Milsmann , Inorg. Chem. 2022, 61, 7338. 35507416 10.1021/acs.inorgchem.2c00182

